# One-Pot Solvothermal Synthetic Route of a Zinc Oxide Nanoparticle-Decorated Reduced Graphene Oxide Nanocomposite: An Advanced Material with a Novel Anticancer Theranostic Approach

**DOI:** 10.1021/acsomega.3c06082

**Published:** 2023-11-28

**Authors:** Nalinee Kanth Kadiyala, Badal Kumar Mandal, L. Vinod Kumar Reddy, Dwaipayan Sen, Sai Kumar Tammina, Crispin H.W. Barnes, Manuel Ñique Alvarez, Luis De Los Santos Valladares, Venkata Subbaiah Kotakadi, Susmila Aparna Gaddam

**Affiliations:** † Trace Elements Speciation Research Laboratory, Department of Chemistry, School of Advanced Sciences, Vellore Institute of Technology (VIT), Vellore 632014, India; ‡ Cellular and Molecular Therapeutics Laboratory, Centre for Biomaterials, Cellular and Molecular Theranostics, Vellore Institute of Technology (VIT), Vellore 632014, India; § Cavendish Laboratory, Department of Physics, University of Cambridge, J.J. Thomson Ave., Cambridge CB3 0HE, U.K.; ∥ Universidad Nacional de Cañete, Jr. San Agustin 124, San Vicente de Cañete15701, Lima, Peru; ⊥ Laboratorio de Ceramicos y Nanomateriales, Facultad de Ciencias Fisicas, Universidad Nacional Mayor de San Marcos, Ap Postal 14-0149, Lima, Peru; # DST PURSE Centre, Sri Venkateswara University, Tirupati 517502, A.P., India; ∇ Department of Virology, Sri Venkateswara University, Tirupati 517502, A.P., India

## Abstract

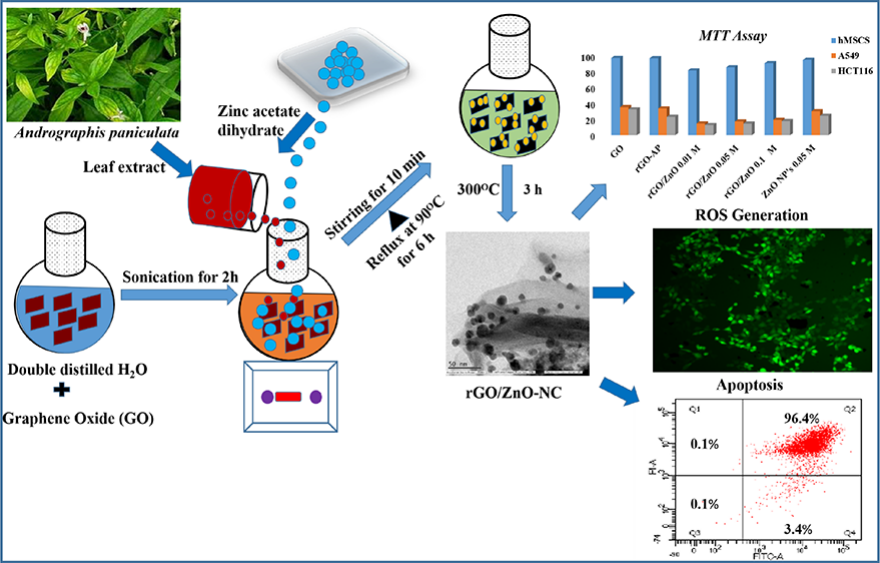

This study focuses on a one-pot solvothermal synthetic route for the preparation of uniformly decorated zinc oxide nanoparticles on the surface of reduced graphene oxide (rGO/ZnO-NC) by using *Andrographis paniculata* leaf aqueous extract as an eco-friendly reducing agent. After characterizing the samples by different physical and chemical techniques, the anticancer activity of the synthesized rGO/ZnO-NC was examined on two human cancerous cell lines (HCT116 and A549) and one normal cell line (hMSCs). The MTT assays revealed that rGO/ZnO-NC exhibited dose-dependent cytotoxicity at a maximum concentration range of 10 ppm and the viability of the cells was drastically decreased to 95–96%. Measurement of reactive oxygen species (ROS) generation and Annexin V-FTIC staining assay revealed that rGO/ZnO-NC induced apoptosis in HCT116 and A549 cell lines. Thus, this study shows that the green-synthesized rGO/ZnO-NC has great potential in developing an efficacious novel therapeutic agent for cancers.

## 1 Introduction

Graphene is one of the rising stars among carbon-based nanomaterials such as fullerenes and carbon nanotubes. It has an interesting and outstanding 2D carbon structure composed of one sp^2^-hybridized carbon atom thick sheet that is tightly packed in a two-dimensional honeycomb lattice. In recent years, graphene has gained considerable research attention, which holds enormous potential when compared to other nanomaterials in the field of nanotechnology, especially in the upcoming fields such as biomedicine,^1^ nanomedicine,^2^ and regenerative medicine.^3^ Graphene holds unique and extraordinary physicochemical features like high thermal, mechanical, and electrical conductivity, optical properties like fast movability of charge carriers, elasticity, huge surface area, biocompatibility, cytocompatibility, and degradability.^4−11^ Moreover, graphene has other promising characteristics such as flexibility in size, highly tunable amphiphilicity, and excellent drug-carrying ability, which promotes design and utilization of low cytotoxicity-based nanomaterials in biomedical applications such as in stem cell and in cancer therapies.^12−15^

Graphene oxide (GO) synthesized by the modified Hummer’s method using graphite as a precursor is highly hydrophilic in nature and thus easy to disperse in solvent medium by adopting a sonication process, which also results in the presence of functional groups like epoxy, carboxyl, and hydroxyl.^16^ These functional groups further play a considerable role in providing active sites for doping metal and metal oxide NPs, which in turn lead to hybridization/functionalization through electrostatic bond interactions.^17,18^ The advancement of graphene chemistry and the evolution of 2D single-layered double-sided graphene sheets into monolithic graphene materials contain three-dimensional (3D) cross-linked structures popularly known as 3D graphene-based gels (3D GBGs), which include hydrogels and aerogels. These materials possess unique hierarchical pore systems such as micro-, meso-, and macro-scale pores in contrast to the 2D graphene films, and in turn, these hierarchical 3D GBG porous structures act as ideal scaffolds and further inhibit the stacking or aggregation phenomenon. Enormous research interest has been devoted toward the fabrication of various combinations of metal and metal oxide 3D GBG moieties such as TiO_2_/reduced graphene oxide aerogel (GA), TiO_2_-graphene hydrogel (TGH), AgBr/graphene aerogel (AgBr/GA), rGH-AgBr@rGO), MoS_2_/P25/graphene aerogel, 3D RGO/Mn_3_O4 aerogel, and 3D rGO/Ti_3_C_2_T_*x*_ (representative of MXenes) hydrogel; in all these combinations, the GO precursor acts as a central part of the moiety. The presence of the above various combinations of 3D GBG molecules are quite applicable in a wide scope of day-to-day life applications such as effective photocatalytic degradation and control of pollutants such as organic pollutants, bacterial pollutants, heavy metal ions, and gaseous pollutants that mainly exist in the water and air medium.^19^ Hence, GO acts as an excellent precursor as well as building block material for producing a variety of nanocomposites for biomedical applications, in view of the increasing demand for the design/development of biomedical and consumer caring products based on graphene nanocomposites and their exposure toward humans and the environment, and also the advancements in synthesizing methodologies and characterization techniques with effective production/availability of these materials in the markets at lower costs.

Inorganic nanoparticles (NPs), including metal oxides, act as great promising materials for applications in medicine fields, i.e., drug/gene delivery, biosensing, cell imaging, and cancer therapy.^20−26^ Among the various types of metal oxide NPs, zinc oxide (ZnO) NPs have gained much research attention due to their low cost, high effectiveness, being easy to fabricate, and applications in diverse fields such as electronics, paints, clothing, sunscreen lotions, and cosmetic products.^27−29^ As the production of ZnO NPs is continuously growing, there is current interest in their utilization in biological research fields like biosensing, nanomedicine, and cancer therapy.^30,31^ It has been demonstrated that ZnO NPs are able to generate cytotoxicity in mammalian cells.^32,33^

Cancer is a serious heterogeneous and complex disease. It is mainly caused by the loss of control over cell proliferation rate capacity/ability by normal cells, which get converted into cancerous cells and spread all over by undergoing three stages of phenomenon, namely, initiation, promotion, and progression. In this sense, the ultimate aim of ZnO NPs is to induce controlled toxicity to kill cancerous cells. To name it, apoptosis is the basic phenomenon in cancer advancement. However, the biggest problem of cancer therapy is that cancerous cells have the ability to propagate by avoiding the apoptosis process, and thus, it becomes a primary objective in curing cancer.

ZnO is a semiconductor material with a wide bandgap energy (∼3.36 eV), and it is also known to be a biocompatible and cost-effective inorganic material. It possesses a huge surface area and strong active surface sites that are readily suitable for doping. ZnO NPs can further improve/restrict the stacking nature of graphene layers through collective van der Waals forces, which can improve the anticancer performance.^34^

Various methods have been pursued for the preparation of uniformly distributed ZnO NPs on the surface of reduced graphene oxide (rGO/ZnO-NC) including microwave,^35^ ultrasound,^36^ and hydrothermal methods,^37^ as well as use of hazardous chemical agents.^38^ However, the reported synthesis methods for the preparation of rGO/ZnO-NC require long reaction rates, high temperature, and high pressure and they release unwanted byproducts. Usually, ZnO NPs are first synthesized separately and then clubbed with a GO solution as a delimiting factor for larger-scale synthesis. A more desirable approach is one-pot synthesis, which has simple steps, is cost-effective, and avoids high-temperature conditions. In this way, to replace the usage of hazardous chemical reagents, many biocompatible and eco-friendly reducing agents have been introduced for the reduction of GO to rGO, such as vitamins,^39^ melatonin,^40^ humanin,^41^ and plant extracts.^42,43^

In a previous work, we reported a one-pot solvothermal method to prepare rGO decorated with zirconia NPs and cytotoxic test in two cancerous cells (A549 and HCT116 cell lines) and one normal human cell line (hMSCs).^44^ At that time, the results indicated that the ZrO_2_/rGO nanocomposites exhibit an apoptosis mechanism in controlling the cancerous cells. The Annexin V-FTIC staining assay technique revealed an 86.9% apoptotic rate, and the MTT assay test results indicated that the % cell viability ratio drastically decreased up to 96–98% at an optimum concentration of 10 ppm whereas in the case of normal cell lines (hMSCs), it did not show any cytotoxic effect. Moreover, from the reactive oxygen species (ROS) analysis, these nanocomposites showed an ∼14.5-fold efficiency rate in HCT116 cell lines when compared to the standard control drug cisplatin. Thus, the ZrO_2_/rGO nanocomposites are highly efficient toward HCT116 cell lines in comparison to A549 cell lines. In this work, we employ an *Andrographis paniculata* aqueous leaf extract as a novel and cost-effective reducing and capping agent to produce uniformly decorated ZnO NPs on the surface of reduced graphene oxide (rGO/ZnO-NC). *A. paniculata* belongs to the family Acanthaceae, and it is generally called *Nila-vembu* in India. It is a well-known plant and found to a larger extent all over India, China, Southeast Asia, and Sri Lanka.

This herb is also popularly known as “Maha-tita” (king of bitters). Regardless of its bitter taste, this specie possesses a broad range of pharmacological activities such as antimicrobial, anti-inflammatory, antioxidant, hypoglycemic, antihyperglycemic, antiallergic, and anticancer.^45,46^ The leaf extract consists of active constituents such as flavonoids, glycosides, steroids, terpenoids, gums, tannins, saponins, stigma sterols, and phenolic compounds.^47^ These active constituents, which may facilitate the green reduction of GO and ZnO precursors, occur at the same time leading to the formation of rGO/ZnO-NC products. According to our literature survey, no work has been published up to now on the anticancer activity of as-prepared uniformly decorated ZnO NPs on the surface of reduced graphene oxide (rGO/ZnO-NC) prepared by using *A. paniculata* aqueous leaf extract. Hence, this is the first ever investigation regarding the green synthesis of rGO/ZnO-NC by a rapid phytochemically assisted solvothermal process. To date, there is no information on the anticancer activity of rGO/ZnO-NC, or the underlying mechanisms of apoptosis in cancer cells induced by dissolution of ZnO NPs from the rGO surface. The present study deals with the design and systematic investigation of the cytotoxicity nature of well-characterized rGO/ZnO-NC toward two human cancerous cells A549 (lung) and HCT116 (colorectal) cell lines) and normal cell lines human umbilical cord blood-derived mesenchymal stem cells (hMSCs). By applying synthesized rGO/ZnO-NC to reduce the cancerous cell counts, the decrease in viability of cells and apoptosis induced by these nanocomposites were thoroughly analyzed by using the MTT assay and flow cytometry analysis, respectively.

## 2 Methods

The details on materials and methods are provided in the Supporting Information.

### 2.1 Synthesis of rGO/ZnO-NC and rGO-AP

GO was successfully synthesized from graphite powder by employing a modified Hummer’s protocol.^48,16,49^ GO acted as a starting material for the synthesis of rGO-AP and rGO/ZnO-NC. The one-pot solvothermal green synthetic route was administered by using the *A. paniculata* leaf extract for preparation of rGO/ZnO-NC and rGO-AP. GO was placed in water (100 mL, 1 mg mL^–1^) and sonicated for 2 h to prepare a homogeneous dispersion. Then, Zn (CH_3_COO_2_)·2H_2_O was added subsequently to the above solution mixture with constant stirring using a magnetic stirrer for about 10 min. After thorough dissolution of the GO–zinc acetate mixture, the final solution’s pH was adjusted to 9.0 by using NaOH (1 M) solution. Then, 10 mL of aqueous *A. paniculata* leaf extract was transferred with the aid of a peristaltic pump and the resultant reaction mixture was heated at 90 °C under reflexions for 6 h to complete reduction of GO. Finally, the mixture was cooled to room temperature. The obtained gray-colored rGO/ZnO-NC were centrifuged, repeatedly washed with double-distilled water, and dried in an oven overnight at 80 °C. Different doping concentrations of Zn(CH3COO2)·2H2O (0.01, 0.05, and 0.1 M) were used in the synthesis process, and the series of products obtained were labeled as rGO/ZnO-NC 0.01 M, rGO/ZnO-NC 0.05 M, rGO/ZnO-NC 0.1 M, and ZnO NPs 0.05 M after calcination at 300 °C for about 3 h at a constant heating rate of 1 °C min^–1^ under ambient conditions. To obtain pure ZnO NPs, the precursor Zn(CH_3_COO_2_)·2H_2_O (0.05 M) was taken alone without GO and followed the same synthesis method used for rGO/ZnO-NC. To investigate the better reduction of GO nanosheets to rGO nanosheets by the *A. paniculata* leaf extract during the formation of rGO/ZnO-NC, rGO-AP alone was synthesized without Zn (CH_3_COO_2_)·2H_2_O by the plant extract. Detailed characterizations of rGO/ZnO-NC were carried out, which are provided in the Supporting Information.

### 2.2 Cell Culture and Cytotoxicity Evaluation/MTT Assays

Cell culture studies and cell viability assays were performed according to the MTT assay, and the detailed methodology is explained in the Supporting Information.^50^

### 2.3 Cytotoxicity Assay–Flow Cytometry

A cytotoxicity assay was performed according to the protocol of Reddy and Sen.^51^

## 3 Results

### 3.1 Characterization of rGO/ZnO-NC

**Figure 1:**
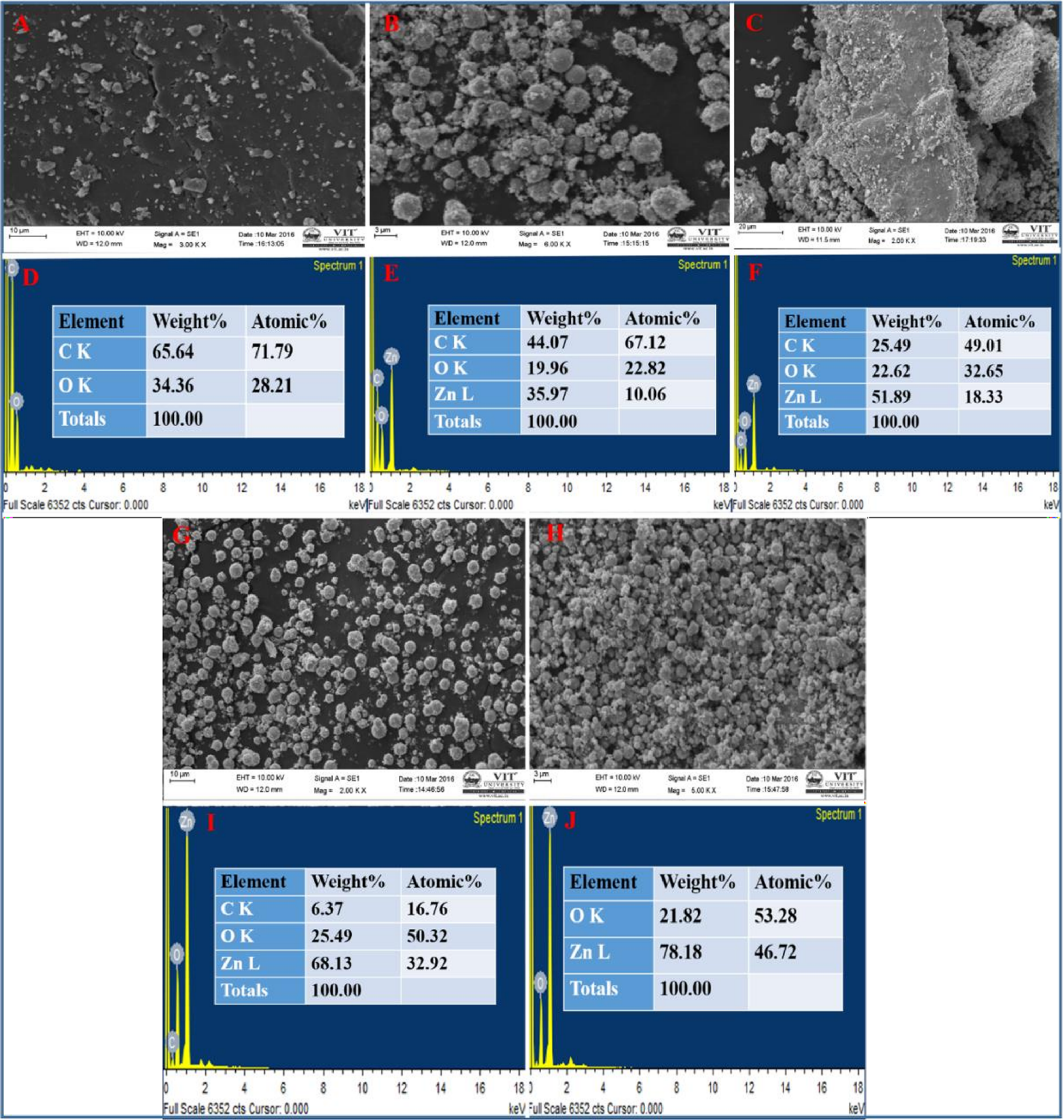
SEM images of rGO-AP (A), rGO/ZnO-NC 0.01 M (B), rGO/ZnO-NC 0.05 M (C), rGO/ZnO-NC 0.1 M (G), and ZnO NP 0.05 M (H). EDS patterns of rGO-AP (D), rGO/ZnO-NC 0.01 M (E), rGO/ZnO-NC 0.05 M (F), rGO/ZnO-NC 0.1 M (I), and ZnO NPs 0.05 M (J).

The surface morphologies of the as-synthesized rGO-AP nanosheets and rGO/ZnO-NC with varying Zn^2+^ ion concentrations fabricated via the green synthetic route were examined thoroughly using SEM analysis. The SEM images are presented in Figure 1. Figure 1A shows that the rGO-AP nanosheets exhibit curled and corrugated morphology. This appearance is intrinsic from the nature of rGO nanosheets, and hence, these 2D graphene sheets are able to thermodynamically stabilize by undergoing bending.^52^Figure 1B,C,G clearly displays ZnO NPs that are uniformly distributed and firmly anchoring to the wrinkled surface of the rGO nanosheets leading to the good combination of rGO/ZnO-NC formation. Note that with an increase in the Zn^2+^ ion concentration, the ZnO NP loading capacity and size on the rGO nanosheets significantly increase. The diameter of ZnO NPs is 100 nm, while in some regions, bigger secondary sphere-shaped NPs (>1 μm) were also noticed, which may be due to formation of agglomerations (Figure 1H). The rGO-AP and rGO/ZnO-NC samples were examined by using EDS (energy-dispersive X-ray spectroscopy) to determine the elemental composition. Figure 1 also presents the EDS patterns of the recorded samples (Figure 1D,E,F,I,J).

The main elements like C, O, and Zn were detected, and no other unexpected elements were observed in the synthesized material, indicating the purity of the materials. Their respective weight and atomic percentages are depicted in Table 1. These results demonstrate that upon increasing the Zn^2+^ ion concentration, the weight fraction of the ZnO NP concentration also increases in the respective samples, i.e., it is 35.97, 51.89, and 68.13% in rGO/ZnO-NC 0.01 M, rGO/ZnO-NC 0.05 M, and rGO/ZnO-NC 0.1 M, respectively (Table 1). Moreover, it can be observed from the EDX analysis data that with an increase in Zn^2+^ ion doping concentration on to the surface of rGO, the carbon content (wt %) ratio of rGO was gradually decreased; the recorded values are 44.07, 25.49, and 6.37% for rGO/ZnO-NC 0.01 M, rGO/ZnO-NC 0.05 M, and rGO/ZnO-NC 0.1 M, respectively (Table 1).

**Table 1:** Elemental (“C”, “O”, and “Zn”) Weight % and Atomic % of the Respective Nanocomposites rGO-AP, rGO/ZnO-NC 0.01M, rGO/ZnO-NC 0.05M, rGO/ZnO-NC 0.1M, and ZnO NPs 0.05M

sample	carbon (wt %)	carbon (at %)	oxygen (wt %)	oxygen (at %)	zinc (wt %)	zinc (at %)
rGO-AP	65.64	71.79	34.36	28.21		
rGO/ZnO-NC 0.01M	44.07	67.12	19.96	22.82	35.97	10.06
rGO/ZnO-NC 0.05M	25.49	49.01	22.62	32.65	51.89	18.33
rGO/ZnO-NC 0.1M	6.37	16.76	25.49	50.32	68.13	32.92
ZnO NPs 0.05M			21.82	53.28	78.18	46.72

**Figure 2:**
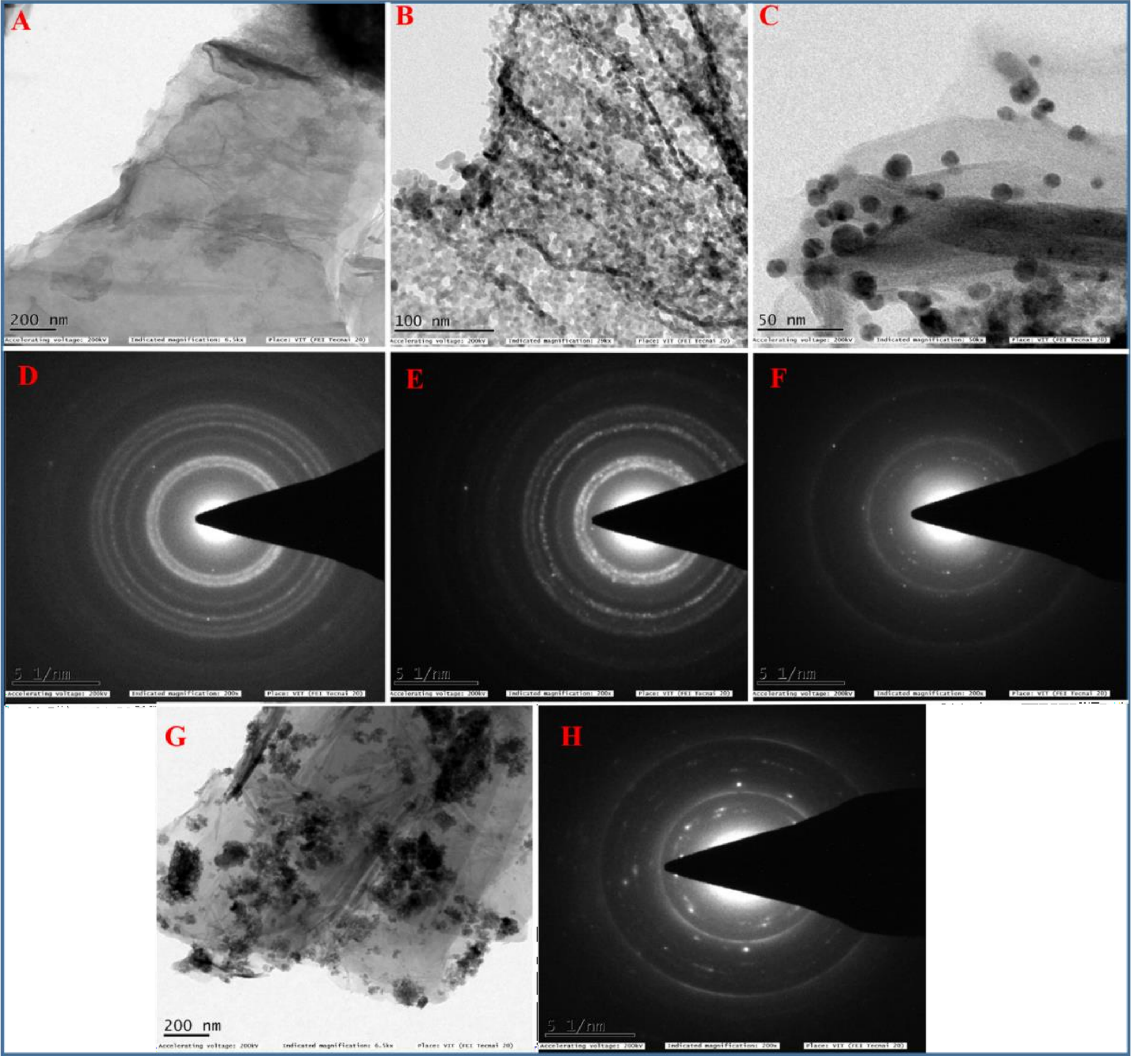
HR-TEM images of rGO-AP (A), rGO/ZnO-NC 0.01 M (B), rGO/ZnO-NC 0.05 M (C), and rGO/ZnO-NC 0.1 M (G). SAED patterns of rGO-AP (D), rGO/ZnO-NC 0.01 M (E), rGO/ZnO-NC 0.05 M(F), and rGO/ZnO-NC 0.1 M (H).

Further morphological and structural characterizations of the as-prepared rGO-AP and rGO/ZnO-NC are depicted in Figure 2. Figure 2A displays the typical TEM image of rGO-AP with a wrinkled transparent paper-like structure, which confirms the exfoliation into a single- or few-layered graphene nanosheets. Figure 2B,C,G presents the TEM images of rGO/ZnO-NC with varying Zn^2+^ ion concentrations, where ZnO NPs are uniformly distributed and anchored firmly onto the surface of rGO nanosheets. Figure 2 demonstrates excellent adherence between rGO and ZnO NPs. Note that functional oxygen groups such as hydroxyl, carboxylic, and epoxy groups exist on the surface of rGO sheets, which provides a free space for the formation of electrostatic interactions between the positively charged Zn^2+^ ions and the negatively charged GO sheets in the precursors, which in turn leads to the firm adhesion of the ZnO NPs onto the rGO sheets in the formed final products.^53^

The selected area electron diffraction (SAED) patterns of rGO-AP and rGO/ZnO-NC are shown in Figure 2D,E,F,H. Figure 2D illustrates the SAED pattern of rGO-AP, and the crystalline carbon is composed of discontinuous dotted circles, which is the characteristic feature of graphene layers with typical hexagonal symmetry. Figure 2E,F,H shows the corresponding SAED patterns of the rGO/ZnO-NC with four bright Debye diffraction rings consistent with the (100), (002), (101), and (102) reflecting planes, respectively. The SAED pattern also confirms that the formed ZnO NPs were in the wurtzite phase.

**Figure 3:**
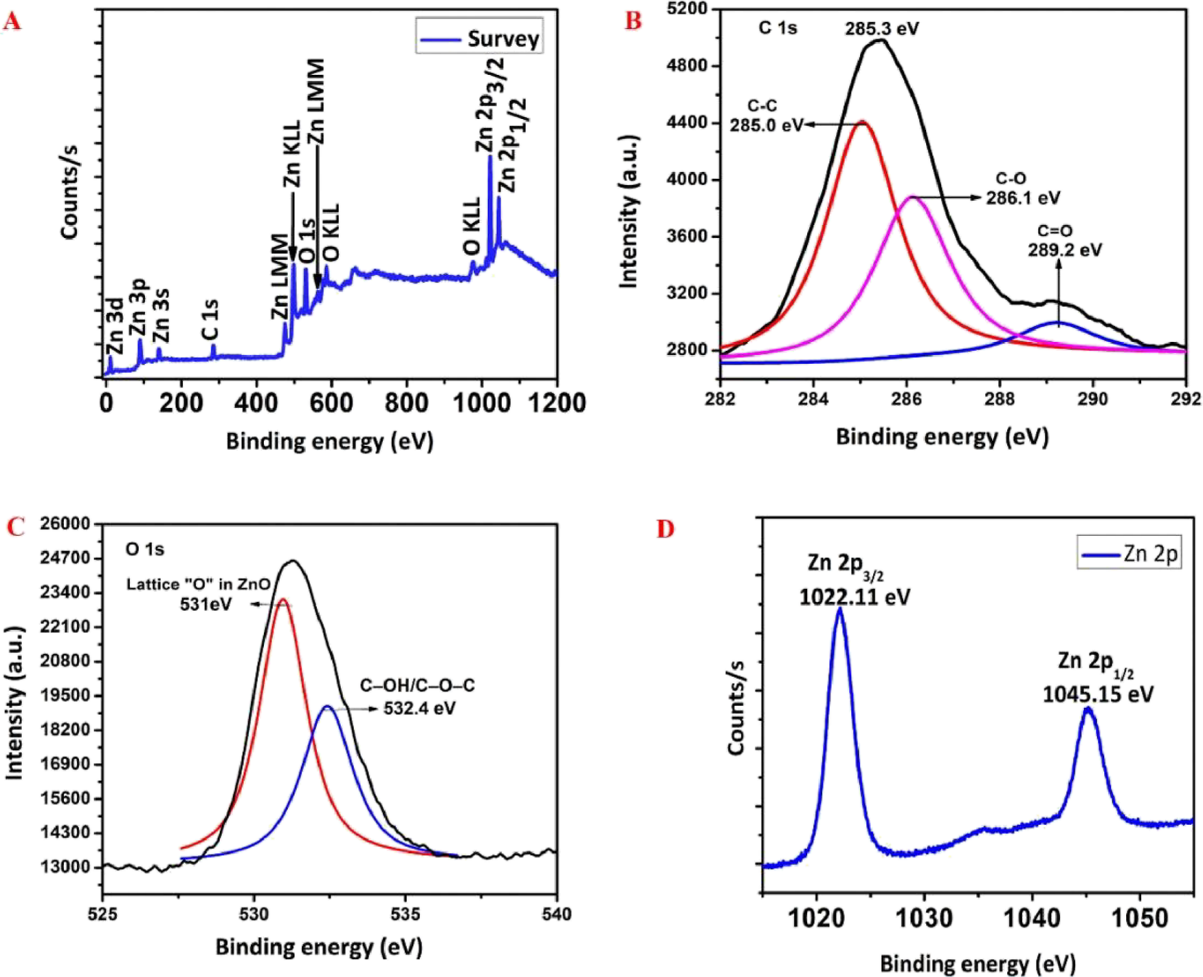
XPS survey spectra (A), C 1s region (B), and the O 1s region (C), and high-resolution spectra of the Zn 2p region for rGO/ZnO-NC 0.05 M (D).

X-ray photoelectron spectroscopy (XPS) analysis was performed to determine the chemical oxidation states of elements present on the surface of rGO/ZnO-NC 0.05 M (Figure 3). Figure 3A presents the complete survey spectrum of the composite, revealing that it is composed of C, O, and Zn elements without peaks related to any other elements. Figure 3B depicts the deconvoluted C 1s XPS spectra with three peaks at 285.0, 286.1, and 289.2 eV. The C–C bond (sp^2^) of graphene corresponds to the binding energy value at 285.0 eV; the peaks located at 286.1 and 289.2 eV ascribe to the C–O and C=C bonds, respectively.^54^ Note that the 0.05 M O 1s profile of rGO/ZnO-NC (Figure 3c) is asymmetric, indicating the existence of two kinds of “O” species in the rGO/ZnO-NC. The first peak at 531.0 eV is originated by the lattice oxygen of ZnO; the second peak at 532.4 eV relates to the C–O–C/C–OH oxygen groups of rGO/ZnO-NC. Figure 3d shows the high-resolution scan of the Zn_2p_ spectrum of rGO/ZnO-NC with two bands at 2p_3/2_ = 1022.11 and 2p_1/2_ = 1045.15 eV, which correlates with the elemental state of Zn^2+^. All of these observations, together with those given in the Supporting Information, further confirm the successful preparation of rGO/ZnO-NC.

### 3.2 Effect of rGO/ZnO-NC on Viability of Cancerous Cells

**Figure 4:**
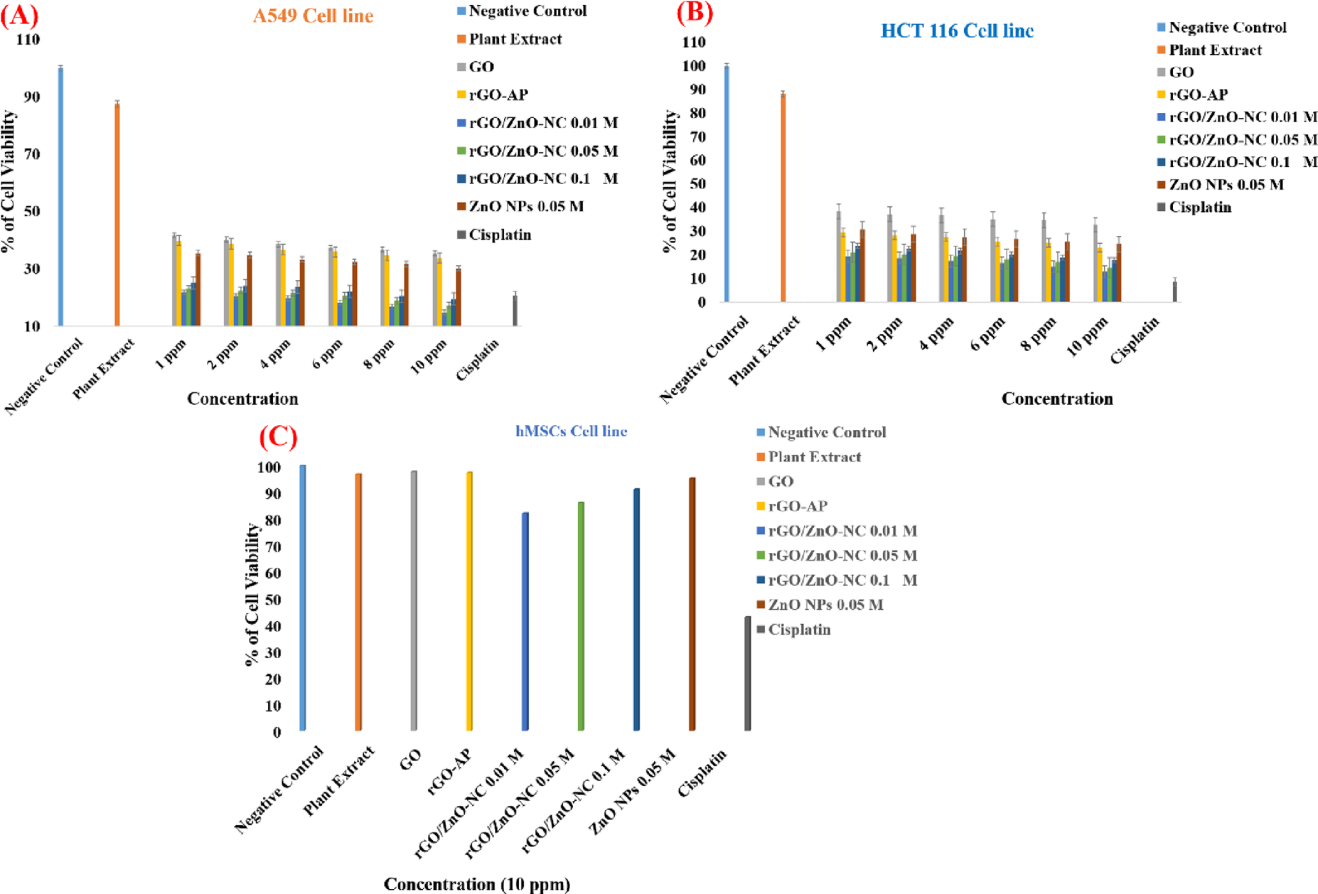
Cytotoxicity effect of GO, rGO-AP, rGO/ZnO-NC 0.01 M, rGO/ZnO-NC 0.05 M, rGO/ZnO-NC 0.1 M, and ZnO NPs 0.05 M on A549 cancer cell lines (A); HCT 116 cell lines (B); and normal cell lines hMSCs (C). All the studied nanomaterials used an optimal concentration of 10 ppm. Data is expressed as mean ± SE of three independent runs for each item (*p < 0.05).

Cell viability of human cancer A549 and HCT116 cell lines was evaluated by the MTT assay and flow cytometry after a 24 h exposure of cell lines with varying concentrations of rGO/ZnO-NC, as described in Section 2. MTT results demonstrate that cytotoxicity was increased with increasing dose of nanocomposites for both the cell lines (Figure 4).

#### 3.2.1 Cell Viability of A549 Cells after Exposure to rGO/ZnO-NC

The plant extract has shown high cell viability of 87.5%, whereas GO and rGO-AP have shown significant reduction in cell viability depending on doses 1, 2, 4, 6, 8, and 10 ppm (41.7 to 35.4% for GO and 39.9 to 33.8% for rGO-AP on the A549 cancer cell line, respectively, *p < 0.05; see Figure 4). The cell viabilities of the A549 cancer cell line are 21.8, 20.5, 19.7, 18.2, 16.75, and 14.8% after exposure to rGO/ZnO-NC 0.01 M; 23.1, 22.5, 21.6, 20.75, 18.9, and 17.3% for rGO/ZnO-NC 0.05 M; and 25.2, 24.1, 23.7, 22.1, 20.5, and 19.4% for the rGO/ZnO-NC 0.1 M exposure, which suggest decreased cell viability with increasing dose of rGO/ZnO-NC (see Figure 4). At a maximum concentration of 10 ppm, rGO/ZnO-NC has shown higher cytotoxicity (19.4%) than the positive control cisplatin (20.6%). With ZnO NP treatment, 35.5, 34.8, 33.3, 32.4, 31.7, and 30.2% of A549 cancer cell viability were observed for 1, 2, 4, 6, 8, and 10 ppm, respectively (see Figure 4A).

#### 3.2.2 Cell Viability Effect of rGO/ZnO-NC on HCT116 Cells

The cell viabilities of HCT116 colon cancer cells are 38.4, 37.3, 63.6, 35.1, 34.5, and 32.7 and 29.4, 28.2, 27.6, 25.4, 24.9, and 23.1% for the 1, 2, 4, 6, 8, and 10 ppm concentrations of GO and rGO-AP, respectively. For ZnO NPs, the viabilities are 30.7, 28.75, 27.4, 26.6, 25.4, and 24.5% for the same concentrations (see Figure 4B). Human HCT116 colon cancer cells show higher cytotoxicity when compared to human A549 cancer cells after exposure to rGO/ZnO-NC (Figure 4). MTT results confirm that the cell viabilities of human HCT116 colon cancer cell lines are 19.3, 18.6, 17.4, 16.5, 14.9, and 12.8% after exposure to 1, 2, 4, 6, 8, and 10 ppm, respectively, of rGO/ZnO-NC 0.01 M, which is significantly reduced (*p < 0.05). The cell viabilities of human HCT116 colon cancer cell lines are 21.1, 20.2, 19.5, 18.3, 16.9, and 14.6%, respectively, after exposure to rGO/ZnO-NC 0.05 M for the same set of concentrations. However, the viabilities are 23.7, 22.5, 21.6, 20.3, 18.8, and 17.8%, respectively, for the same set experiments after exposure to rGO/ZnO-NC 0.1 M. Interestingly, the cell viability is slightly higher with exposure to rGO/ZnO-NC prepared from higher doping of rGO with ZnO NPs (see Figure 4) due to slightly higher hydrodynamic sizes.

#### 3.2.3 Cytotoxic Effect of rGO/ZnO-NC on Normal Cells (hMSCs)

Along with cancer cells, the cytotoxic effect of the synthesized nanomaterials was also tested on normal cells (hMSCs) at appropriate concentrations. There is no significant toxicity of the nanocomposites on the hMSCs (viabilities of 97.7, 97.4, 82.03, 85.99, 91.05, and 95.28% after exposure to GO, rGO, rGO/ZnO-NC 0.01, 0.05, and 0.1, and ZnO NPs 0.05 M, respectively) (see Figure 4C) when compared with positive control cisplatin (43.07%). These results clearly demonstrate that the one-pot solvothermally synthesized rGO/ZnO-NCs are toxic to cancer cells and are biocompatible to normal cells. The IC_50_ values of GO, rGO-AP, rGO/ZnO-NC (0.01, 0.05, 0.1 M), and ZnO NPs 0.05 M are included in Table S2. The IC_50_ values obtained for HCT116 and A549 cell lines after exposure to rGO/ZnO-NC 0.01 M are 0.0515 and 0.3461 μg L^–1^, respectively, which are lower than the others.

### 3.3 Flow Cytometry Study for Measurement of Cell Viability

**Figure 5:**
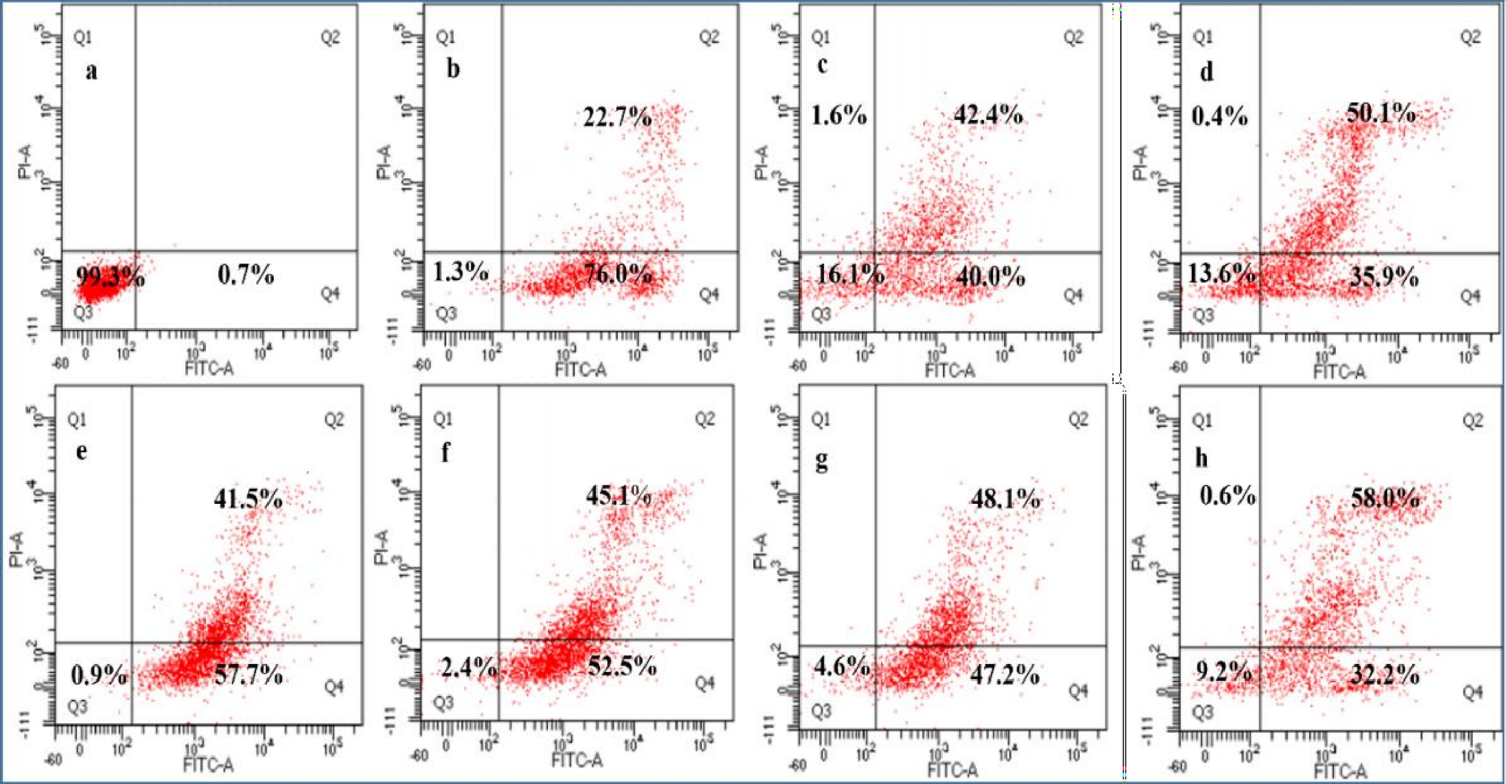
Flow cytometric analysis of apoptotic cell death after treatment with a maximum concentration of 10 ppm for rGO/ZnO-NCs exposed to A549 cell lines for 24 h and double labeling with Annexin V-FITC and PI. Scatter diagrams of cells exposed with respective compounds are shown in (a–h) as follows: negative control (a), positive control drug (cisplatin) (b), GO (c), rGO-AP (d), rGO/ZnO-NC 0.01 M (e), rGO/ZnO-NC 0.05 M (f), rGO/ZnO-NC 0.1 M (g), and ZnO NPs 0.05 M (h). In the flow cytogram, the cells in the Q3 region denotes live cells, Q4: apoptotic, Q2: late apoptotic, and Q1: necrotic cells. Data is representative of three independent experiments.

The flow cytometry results demonstrate that negative control has shown 99.3% human A549 lung cancer cell viability. For the other groups GO, rGO-AP, and rGO/ZnO-NC (0.01, 0.05, 0.1, and ZnO NPs 0.05 M), the cell viabilities are reduced to 16.1, 13.6, 0.9, 2.4, 4.6, and 9.2%, respectively. Positive control cisplatin has shown 1.3% cell viability (see Figure 5 and Tables S3 and S4).

**Figure 6:**
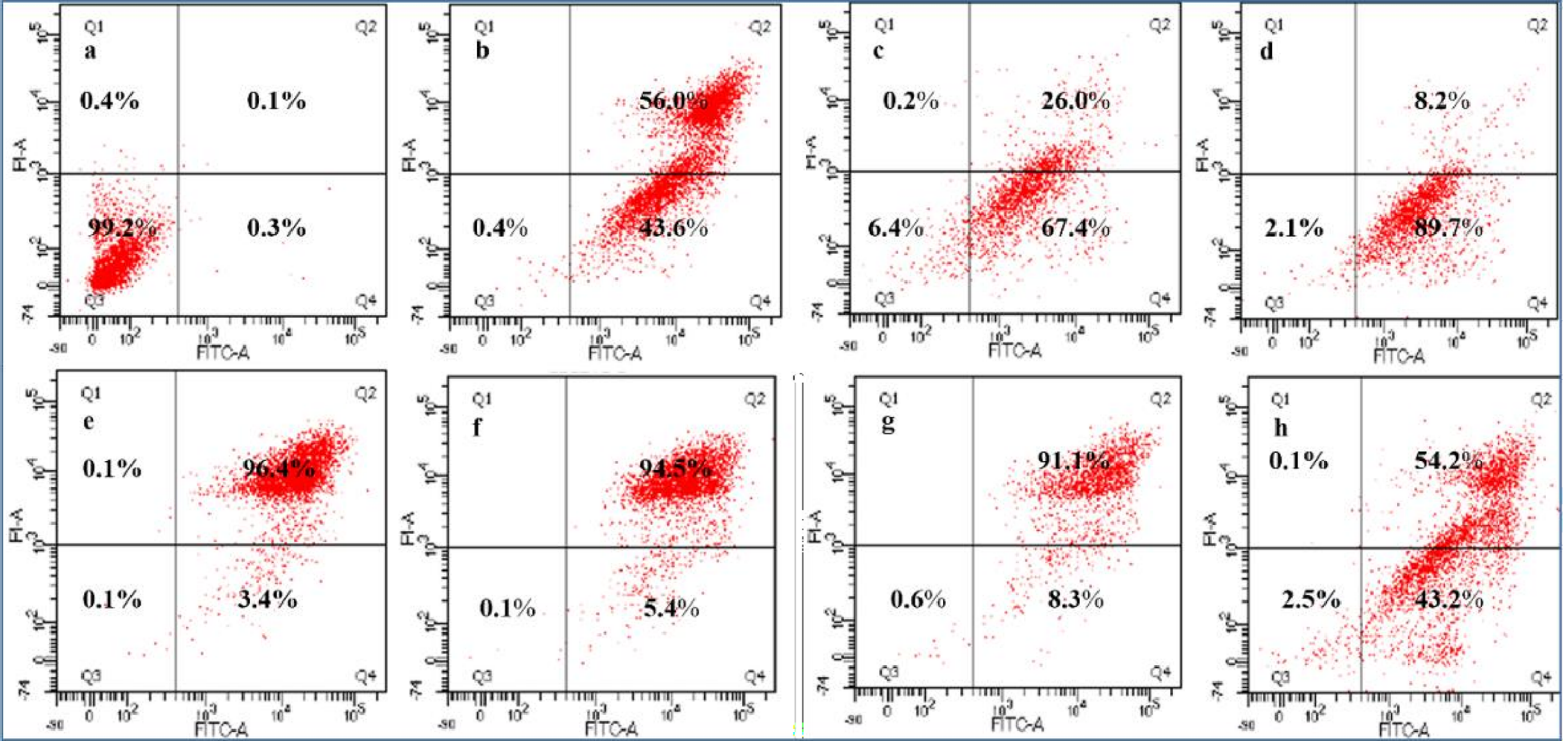
Flow cytometric analysis of apoptotic cell death after treatment with a maximum concentration of 10 ppm for rGO/ZnO-NCs exposed to HCT116 cell lines for 24 h and double labeling with Annexin V-FITC and PI. Scatter diagrams of cells exposed with respective compounds are shown in (a–h) as follows: negative control (a), positive control (cisplatin) (b), GO (c), rGO-AP (d), rGO/ZnO-NC 0.01 M (e), rGO/ZnO-NC 0.05 M (f), rGO/ZnO-NC 0.1 M (g), and ZnO NPs 0.05 M (h). In the flow cytograms, the cells in the Q3: region denote live cells, Q4: apoptotic, Q2: late apoptotic, and Q1: necrotic cells. Data is representative of three independent experiments.

The flow cytometry results demonstrate that negative control presents 99.2% human HCT116 colon cancer cell viability. Other groups, i.e., GO, rGO, rGO/ZnO-NC (0.01, 0.05, and 0.1 M and ZnO NPs 0.05 M), show viability of 6.4, 2.1, 0.1, 0.1, 0.6, and 2.5%, respectively, whereas positive control (cisplatin) presents 0.4% cell viability (see Figure 6 and Table S5). Based on the above results, the optimal concentration is 10 ppm (it shows the highest cytotoxicity) and thus used for further experiments (see Figure 4).

### 3.4 Role of rGO/ZnO-NCs on ROS Generation

**Figure 7:**
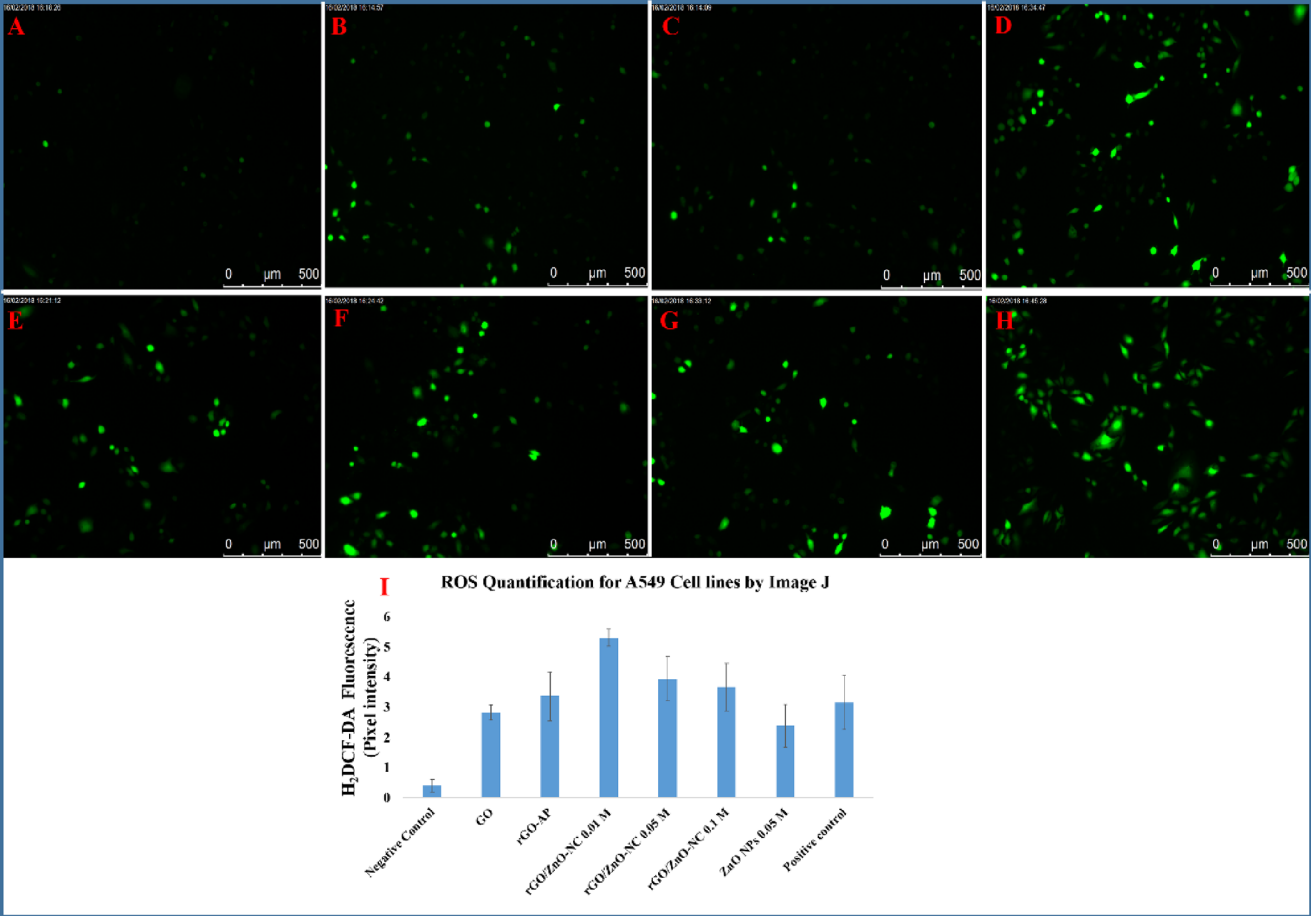
Representative fluorescence microscopic images of ROS generation for A549 cancerous cell lines: negative control (A), GO (B), rGO-AP (C), rGO/ZnO-NC 0.01 M (D), rGO/ZnO-NC 0.05 M (E), rGO/ZnO-NC 0.1 M (F), ZnO NPs 0.05 M (G), and positive control drug (cisplatin) (H). Images are representative of three independent experiments.

**Figure 8:**
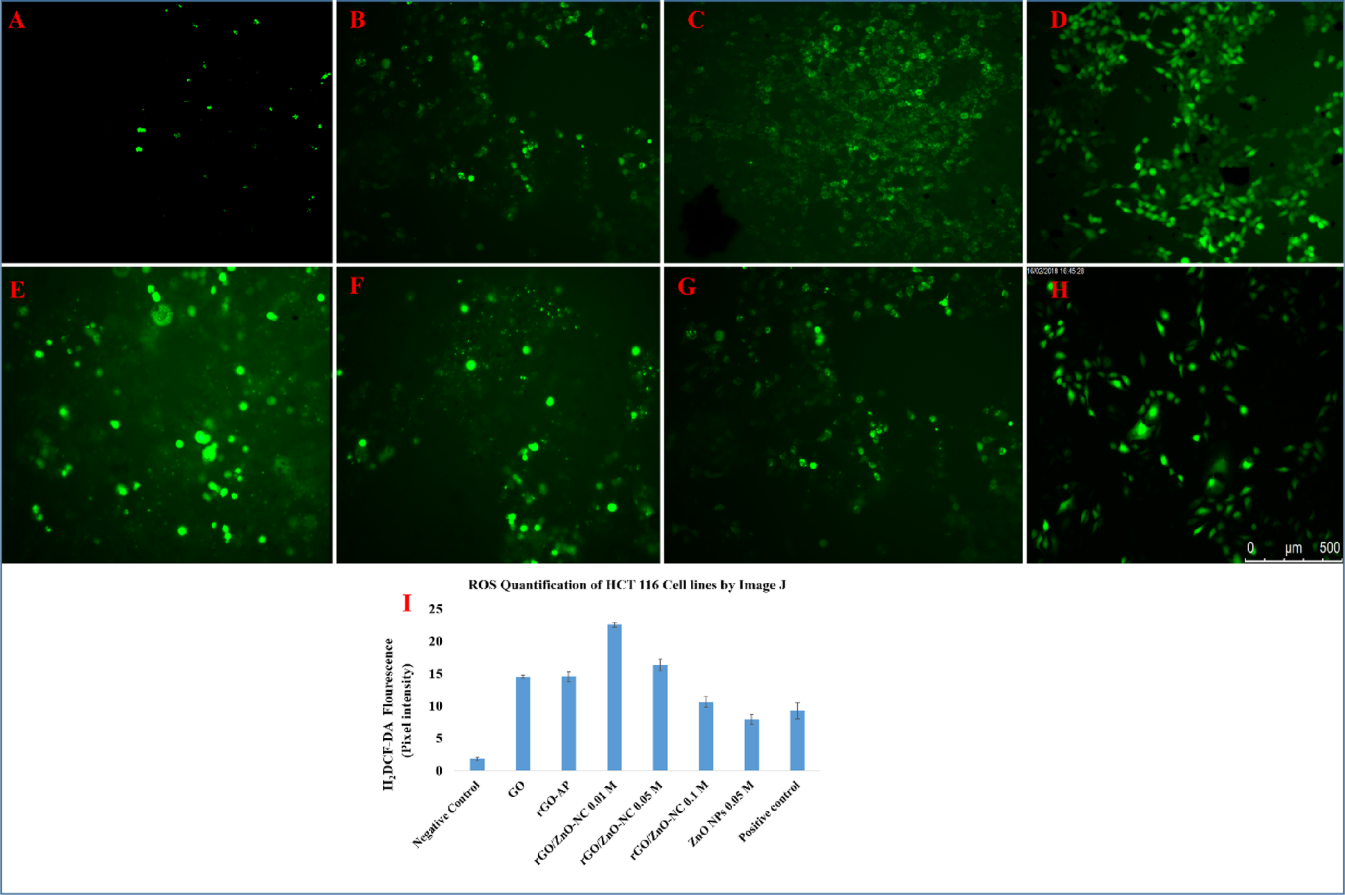
Representative fluorescence microscopic images of ROS generation for HCT116 cancerous cell lines: negative control (A), GO (B), rGO-AP (C), rGO/ZnO-NC 0.01 M (D), rGO/ZnO-NC 0.05 M (E), rGO/ZnO-NC 0.1 M (F), ZnO NPs 0.05 M (G), and positive control drug (cisplatin) (H). Images are representative of three independent experiments. Quantification of the mean fluorescence intensity using ImageJ from three images from each run from different groups. Data is average ± SE of three independent runs done in triplicate wells in each run (I). *p < 0.05.

Several reports have highlighted that different nanomaterials tend to initiate oxidative stress by means of generating ROS, which results in induced cytotoxicity^55,56^ The oxidative stress potentials of the as-synthesized materials GO, rGO-AP, rGO/ZnO-NC (0.01, 0.05, 0.1 M), and ZnO NPs 0.05 M were evaluated against A549, HCT116, and human cell lines (hMSCs). Similar to the results of the cell viability studies, the higher generation of ROS levels and cellular oxidative stress are found in the order of rGO/ZnO-NC 0.01 M > rGO/ZnO-NC 0.05 M > rGO/ZnO-NC 0.1 M > ZnO NPs 0.05 M > rGO-AP > GO. For the hMSC cells, the ROS generation in the HCT116 cell lines is higher than that in the A549 cell lines (see Figures 7 and 8).

ImageJ quantification analysis demonstrates a significant increase of ROS by ∼3.5-, ∼4-, ∼6-, ∼7.5-, ∼8-, and ∼10-fold for GO, rGO-AP, ZnO NPs 0.05 M, rGO/ZnO-NC 0.1 M, rGO/ZnO-NC 0.05 M, and rGO/ZnO-NC 0.01 M, respectively. When compared with standard drug cisplatin, the synthesized rGO/ZnO-NC 0.01 M exhibits ∼3.5-fold increased ROS generation. In the case of HCT116 cells, ROS generation is increased by ∼14.5-, ∼14.5-, ∼23-, ∼15-, ∼10-, ∼6.5-fold for rGO/ZnO-NC 0.01 M, rGO/ZnO-NC 0.05 M, rGO/ZnO-NC 0.1 M, ZnO NPs 0.05 M, and rGO-AP and GO, respectively (see Figure 8). Compared to cisplatin, rGO/ZnO-NC 0.01 M shows increased ROS generation by ∼14.5-fold, where the mean fluorescent intensity is compared with the control (see Table S3 and Figures 7I and 8I). The studied nanocomposites did not show any ROS generation in the hMSCs, which demonstrates that no cytotoxic effect is observed in normal cells (Figure S9).

Quantification of the mean fluorescence intensity using ImageJ from three images from each run from different groups (I). Data is average ± SE of three independent runs done in triplicate wells in each run. *p < 0.05.

## 4 Discussion

The advancement of anticancer therapeutic agents enabling to induce both cytotoxicity and apoptosis are interesting approaches for cancer cure and treatment. The present work explores the anticancer therapeutic nature of one-pot solvothermal synthesized ZnO NP decorating the surface of reduced graphene oxide (rGO/ZnO-NC) toward two human cancerous cell lines A549 (lung cancer), HCT 116 (colorectal cancer), and one normal cell line hMSCs (umbilical cord blood derived). The physicochemical characterization of the synthesized rGO/ZnO-NC, performed by using different analytical tools like XRD, FTIR, SEM, TEM, EDS, Raman spectroscopy, XPS spectroscopy, and dynamic light scattering (DLS), determines parameters like crystal structure, functional groups, shape, size, purity, hydrodynamic size, agglomeration, and aqueous stability. The decrease in the XRD peak intensity observed after the reduction of GO to rGO-AP using the aqueous *A. paniculata* leaf extract confirms that the oxygen-containing functional groups existing on the GO surface are effectively removed/reduced. ZnO nanocrystals decorating the rGO surface in the rGO/ZnO-NC are in the form of wurtzite. The FTIR spectroscopy technique reveals that oxygen-containing moieties such as hydroxyl, carboxyl, and epoxy groups were successfully reduced from the GO surface to rGO-AP via biological reduction. SEM and TEM results show that ZnO NPs are mostly spherical in shape and uniformly distributed throughout the surface of the rGO. EDS data indicate that synthesized GO, rGO-AP, and rGO/ZnO-NC are composed of C, O, and Zn as main elements, and no other unexpected elements are observed indicating the high purity of the synthesized nanomaterials. Raman spectroscopy suggests the presence of defects on the carbon surfaces and reestablishment of the numerous conjugated graphene (sp^2^ carbon) networks, which confirms that rGO/ZnO-NC were well established and were composed of pure ZnO NPs decorated on graphene nanosheets. X-ray photoelectron spectroscopy (XPS) predicts the surface chemical oxidation states of elements present in the rGO/ZnO-NC 0.05 M. Once the GO, rGO-AP, and rGO/ZnO-NCs were introduced into biological buffer systems like PBS, cell culture medium, and water, the nanocomposite sizes changed to approximately 5 to 10 times higher when compared to primary size. These changes appeared due to interactions between the rGO/ZnO-NCs and the components present in cell growth media, which have been shown to impact on agglomeration and/or precipitation of these nanocomposites. As a result, it ultimately leads to lesser zeta potential values observed in PBS and cell culture medium when compared to water medium. Cytotoxic studies play a key role in estimating the anticancer levels of any compound. Different nanomaterials have shown different anticancer activity levels. According to the present study, low cytotoxicity levels are observed from the raw components (GO, rGO, and ZnO NPs) compared to different doped rGO/ZnO-NCs (0.01, 0.05, and 0.1 M) and cisplatin. In contrast to the other NCs, rGO/ZnO-NC 0.01 M/0.05 M have shown more anticancer activity than cisplatin at high concentrations (6, 8, and 10 ppm) as 18.2, 16.7, 14.8 vs 20.6%, respectively. Similar results are obtained from the Annexin V assay for rGO/ZnO-NC 0.01 M (A549 vs HCT116: 0.9 vs 0.1%), rGO/ZnO-NC 0.05 M (A549 vs HCT116: 2.4 vs 0.1%), and rGO/ZnO-NC 0.1 M (A549 vs HCT116: 4.6 vs 0.6%), which suggests higher anticancer effect in HCT116 than in A549. These results indicate that the rGO/ZnO-NC present significant (*p < 0.05) cytotoxicity toward both the A549 and HCT116 cancer cells (see Figures 5 and 6).

To find out the statistical significance between two different treatments, we performed a two-tailed paired Student’s *t* test, assuming absolute *t*-value as 2.23 at a degree of freedom (df) of 10 and probability (*p*) of 0.05. We calculated the *t*-value to check the null hypothesis that two treatment groups for A549 cells are not statistically different. The *t*-value of rGO-AP is found 1.29, which is lower than the absolute *t*-value 2.23, and those for rGO/ZnO-NC 0.01 M, rGO/ZnO-NC 0.05 M, rGO/ZnO-NC 0.1 M, and ZnO NPs 0.05 M are 13.95, 13.51, 12.05, and 4.32, respectively, which are higher than 2.23 (see Figure 4 and Table S6A). Hence, the rGO-AP-treated group is not statistically different from the GO-treated group, but all the others are. The Student’s *t* test performed to find out probability (*p*) reveals that all the *p*-values are much lower than 0.05, which suggests that all the treatment groups are statistically significant (see Figure 4 and Table S6A). Similarly, we calculated the *t*-values for all the treated groups for the HCT116 cells. Except for the rGO-AP and rGO/ZnO-NC 0.05 M-treated groups, all the other treated groups are statistically different (see Figure 4 and Table S6B). The Student’s *t* test and probability (*p*) values for all treated groups are lower than 0.05, suggesting high statistical significance (Table S6B), whereas all treated groups with hMSCs are not statistically different (Table S6C) as the *t*-values of all treated groups are lower than the absolute *t*-value, which suggests that the hMSCs cells are not affected due to the exposure with these nanomaterials.

ROS are natural derivatives by cellular oxidative metabolism and play various roles in cellular activities, i.e., cell survival, cell death, differentiation, cell signaling, and inflammation. When the NPs are exposed to the cells, they interact with cell membranes and transfer ionic signals to different cell organelles, which leads to the generation of excessive ROS levels. Eventually, the ROS generation leads to cell death. Moreover, excessive ROS generation leads to oxidative stress, which is a key factor in nanotoxicity-mediated cytotoxicity, DNA damage, apoptosis, and cancer.^57^

Based on several reports on nanotoxicology, both graphene and semiconductive nanomaterials such as ZnO NPs are very much effective to sensitize the elevated level of ROS production within the cells that ultimately results in cell death.^58−60^ Hence, the present work utilized both graphene and ZnO NPs decorated on rGO nanocomposites to test their anticancer activity. The molecular mechanisms between ROS generation/oxidative stress and NP-mediated cell death are well studied.^61−63^ In brief, the synthesized rGO/ZnO-NC are highly effective in the generation of ROS species within the cancer cells at low or moderate levels, which affects the signal transmission and cell proliferation rates, whereas at high concentration ratios, it leads to the modification of lipids and proteins and damage of DNA. Excessive levels of ROS cause damage to DNA and thereby result in promotion of genomic instability, autophagy, and ultimately cell death.

**Scheme 1:**
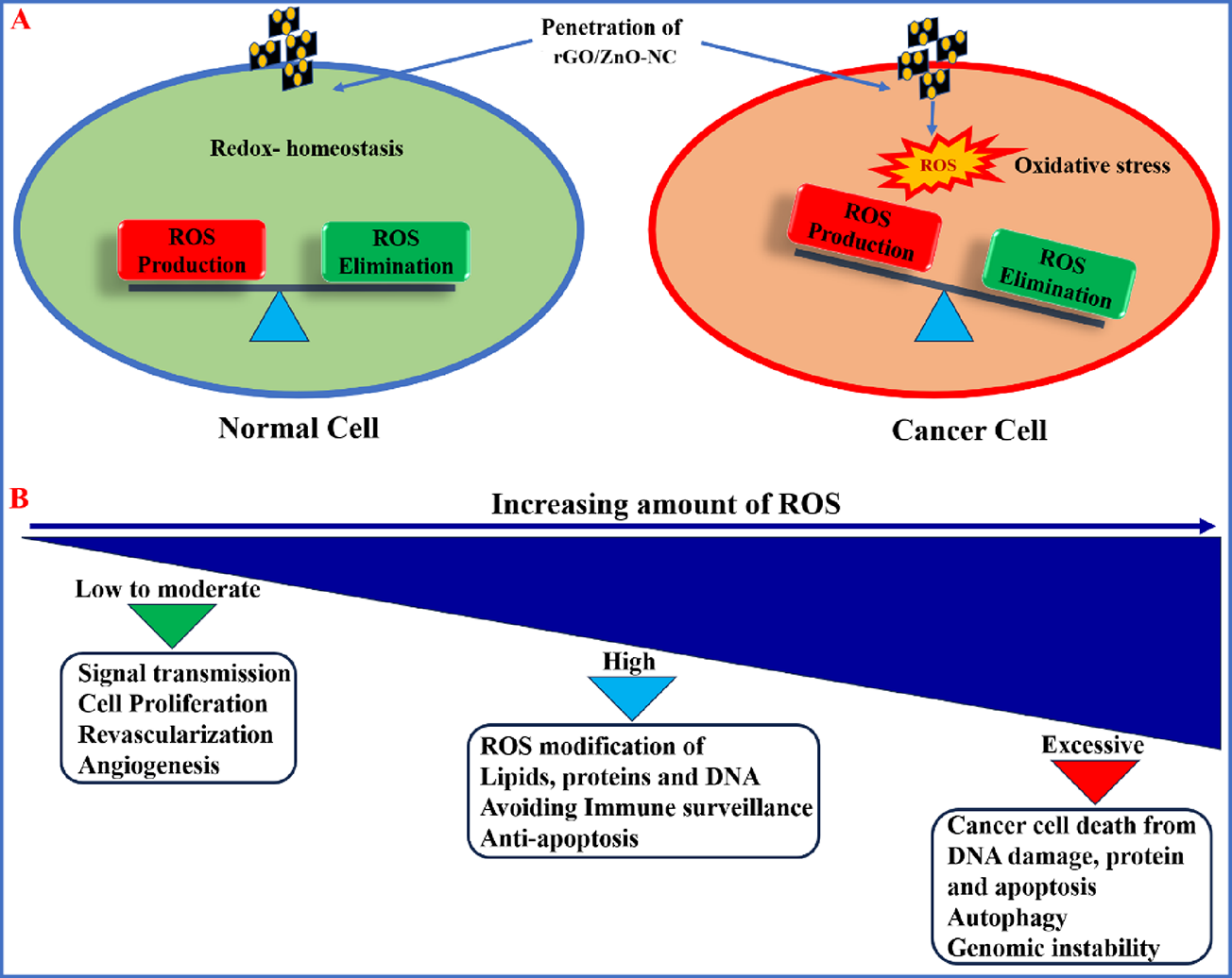
Possible Penetration Processes of rGO/ZnO-NC into the Cells Resulting in the Generation of ROS Species and Their Drastic Effects toward Cancer Cells in Comparison to the Normal Cells

This is a strategic phenomenon that mainly takes place in cancer cells but not in normal cells. Hence, the role of ROS can be considered to be a double sword in the development of cancer therapeutic agents (see Scheme 1). In our study, the generation of ROS drastically increased with nanocomposite treatment. Especially it becomes ∼14-fold higher than the cisplatin in HCT116 cells (Figure 6). The other nanocomposites also generated a significantly increased level of ROS (Figures 7 and 8). The levels of ROS generation represent oxidative stress, which correlates with concentration of nanomaterials exposed to cells.^64^ This correlation exists in the present work at a high concentration (10 ppm); that is, a high level of ROS is generated, which leads to more cancer cell death. All these results demonstrate that the synthesized rGO/ZnO-NC has a good cytotoxic effect toward cancer cells and no toxicity to normal cells. However, in-depth anticancer cellular mechanisms need to be investigated.

The flow cytometry results demonstrate that posttreatment of human A549 lung cancer cells with different nanocomposites increases ROS in the cells, causing programmed cell death, i.e., apoptotic cell death (see Figure 5). The A549 cancer cells exposed to GO, rGO-AP, and rGO/ZnO-NC (0.01, 0.05, and 0.1 M and ZnO NPs 0.05 M) show 40.0, 35.9, 57.7, 52.5, 47.2, and 32.2% apoptotic cell death, respectively. These results suggest that ZnO NPs doped with rGO-NC produced more ROS, resulting in the highest number of apoptotic cell deaths (see Figure 5). Similarly, the HCT116 cancer cells exposed to GO, rGO-AP, and rGO/ZnO-NC (0.01, 0.05, and 0.1 M and ZnO NPs 0.05 M) show 67.4, 89.7, 3.4, 5.4, 8.3, and 43.2% apoptotic cell death, respectively (see Figure 6). Interestingly, the HCT116 cells were less prone to apoptotic cell death compared to A549 cancer cells. However, HCT116 cells were highly prone to late apoptotic cell death (54.2–96.4%) compared to late apoptotic cell death of A549 cells (41.5–58.0%) (Figures 5 and 6). Further studies are necessary to confirm these observations and to understand the actual mechanism of apoptotic cell death by measuring cytochrome C, caspase-3, and caspase-9 activation for early-stage and midstage apoptotic cell death. DNA fragmentation and nuclear collapse could be performed using the TUNEL assay for late-stage apoptotic cell death. This information will be generated in a future project that will diagnose the actual reasons for the different responses of A549 and HCT116 cancer cells treated with NCs during early- and late-stage apoptotic cell death. In addition, Figure S10 shows the fluorescence microscopic images of ROS generation after exposure of the studied different nanomaterials to hMSC normal cells and it is evident that no ROS is generated after the treatment. This result clearly suggests that ZnO NPs doped with rGO-NC and ZnO NPs are nontoxic to hMSC normal cells, confirming its potential applications in biomedical and cancer therapy.

## Conclusions

ZnO NPs on the surface of reduced graphene oxide, rGO/ZnO-NCs, were successfully synthesized by a one-pot solvothermal route using the *A. paniculata* leaf aqueous extract as an eco-friendly reducing agent, and they were characterized by different physical and chemical techniques. The anticancer activity of the synthesized rGO/ZnO-NC was examined on two human cancerous cell lines (HCT116 and A549) and one normal cell line (hMSCs). Based on the current results, the rGO/ZnO-NCs show a distinct anticancer effect toward human HCT116 and A549 cancer cells while it poses no effect on normal cells (hMSCs). The effect is more pronounced and mediated through oxidative stress by ROS generation in a similar manner that the chemotherapeutics drugs induce/trigger apoptosis. Hence, having these exciting potential properties, the rGO/ZnO-NC could serve as a potential anticancer agent in cancer therapy.

## Supporting Information Available

Spectral characterization of different samples; UV–vis spectra of as-prepared nanomaterials; hydrodynamic size distribution and zeta potential of GO; hydrodynamic size distribution and zeta potential of rGO-AP; hydrodynamic size distribution and zeta potential of rGO/ZnO-NC-0.01 M; hydrodynamic size distribution and zeta potential of rGO/ZnO-NC 0.05 M; hydrodynamic size distribution and zeta potential of rGO/ZnO-NC 0.1 M; hydrodynamic size distribution and zeta potential of ZnO NPs 0.05 M; representative fluorescence microscopic images of ROS generation for the HCT116 cancerous cell line; representative fluorescence microscopic images of ROS generation for the hMSC normal cell line; zeta potential and DLS analysis of different nanomaterials; IC_50_ values (μg L^–1^) of different nanomaterials for human cancerous cell lines; ROS quantified mean fluorescence intensity (MFI) data values for different nanomaterials with positive control drug (cisplatin) for human cancerous cell lines; data obtained from FITC-conjugated Annexin V and PI staining assays for A549 cell lines; data obtained from FITC-conjugated Annexin V and PI staining assays for HCT116 cell lines; and statistical analysis of data sets for significant evaluation of anticancer activity of different nanomaterials on A549, HCT116, and hMSC control cell lines (PDF)
