# Supplementary material for: One-Pot Solvothermal Synthetic Route of a Zinc Oxide Nanoparticle-Decorated Reduced Graphene Oxide Nanocomposite: An Advanced Material with a Novel Anticancer Theranostic Approach
Source: ACS Omega. 2023 Nov 28;8(49):46763–76. doi: 10.1021/acsomega.3c06082 (PMC10720013; doi:10.1021/acsomega.3c06082)
Supplement: Supplementary file 1 — ao3c06082_si_001.pdf [file ao3c06082_si_001.pdf]

Supplementary Information

One-pot solvothermal synthetic route of zinc oxide nanoparticles decorated reduced graphene oxide nanocomposite: An advanced material with novel anticancer theranostic approach

Nalinee Kanth Kadiyala,[a] Badal Kumar Mandal,[a,*] L. Vinod Kumar Reddy,[b] Dwaipayan Sen,[b] Sai Kumar Tammina,[a] Crispin H.W. Barnes,[c] Manuel Ñique Alvarez,[d] Luis De Los Santos Valladares,[c,e*] Venkata Subbaiah Kotakadi,[f] Susmila Aparna Gaddam[g]

[a] *Trace Elements Speciation Research Laboratory, Department of Chemistry, School of Advanced Sciences, Vellore Institute of Technology (VIT), Vellore 632014, India*

[b] *Cellular and Molecular Therapeutics Laboratory, Centre for Biomaterials, Cellular and Molecular Theranostics, Vellore Institute of Technology (VIT), Vellore 632014, India*

[c] *Cavendish Laboratory, Department of Physics, University of Cambridge, J.J. Thomson Ave., Cambridge CB3 0HE, UK*

[d] *Universidad Nacional de Cañete, Jr. San Agustin 124, San Vicente, Cañete, Lima, Perú.*

[e] *Laboratorio de Ceramicos y Nanomateriales, Facultad de Ciencias Fisicas, Universidad Nacional Mayor de San Marcos, Ap. Postal 14-0149, Lima, Peru.*

[f] *DST PURSE Centre, Sri Venkateswara University, Tirupati-517502.A.P. India*

[g] *Department of Virology, Sri Venkateswara University, Tirupati-517502.A.P. India*

*Corresponding Authors. Dr. Badal Kumar Mandal, Tel.:+914162202339; Fax: +914162243092; E-mail address: badalmandal@vit.ac.in, badalkmandal@gmail.com and Dr. Luis De Los Santos Valladares, E-mail address: *ld301@cam.ac.uk*

Brief statement

- Spectral characterization of different samples
- UV-vis spectra of as-prepared nanomaterials
- Hydrodynamic size distribution and Zeta potential of GO
- Hydrodynamic size distribution and Zeta potential of rGO-AP
- Hydrodynamic size distribution and Zeta potential of rGO/ZnO-NC 0.01M
- Hydrodynamic size distribution and Zeta potential of rGO/ZnO-NC 0.05M
- Hydrodynamic size distribution and Zeta potential of rGO/ZnO-NC 0.1M
- Hydrodynamic size distribution and Zeta potential of ZnO NPs 0.05M
- Representative fluorescence microscopic images of ROS generation for HCT116 cancerous cell line
- Representative fluorescence microscopic images of ROS generation for hMSCs normal cell line
- Zeta potential and DLS analysis of different nanomaterials
- IC_{50} Values (µg L^{-1}) of different nanomaterials for human cancerous cell lines
- ROS quantified mean fluorescence intensity (MFI) data values for different nanomaterials with Positive control drug (Cisplatin) for human cancerous cell lines
- Data obtained from FITC-conjugated Annexin-V and PI staining assay for A549 cell lines
- Data obtained from FITC-conjugated Annexin-V and PI staining assay for HCT116 cell lines
- Statistical analysis of data sets for significant evaluation of anticancer activity of different nanomaterials on A549, HCT116 and hMSCs control cell lines

Materials and Methods

Materials

Graphite powder (100 mesh, 99.9%, Sigma-Aldrich), Sodium nitrate ($NaNO_3$, AR grade, Qualigens®, India), Potassium permanganate, ($KMnO_4$, >99%, Sigma-Aldrich), sulfuric acid (H_2SO_4, 98% Sigma-Aldrich), Hydrogen peroxide (H_2O_2, 30%, Sigma-Aldrich),

Hydrogen chloride (HCl, 36%, sigma-Aldrich), Zinc acetate dihydrate ($Zn(CH_3COO)_2.2H_2O$, >99%, Sigma-Aldrich), Dulbecco's modified Eagle's medium (DMEM, HI Media), α-minimum essential medium (α-MEM, Gibco/Life Technologies, USA), (Foetal bovine serum (FBS, HI Media), 1% pen/strep (HI Media), 3-(4, 5-dimethylthiazol-2-yl)-2, 5-diphenyltetrazolium bromide (MTT, HI Media), Human umbilical cord blood derived mesenchymal stem cells (hMSCs, PromoCell, Germany), Human colon (HCT116) and lung (A549) carcinoma cell lines (American Type Culture Collection, ATCC; National Sciences for Cell Sciences, NCCS, Pune, India), Drug Cisplat (Cisplatin Injection I.P. 50mg/50mL, Zydus Oncosciences), Double distilled water were used throughout the experiment.

Preparation of Andrographis paniculata leaf extract

Fresh and healthy plant leaves of Nelavemu (*Andrographis paniculata*) were collected from the surroundings of Vellore Institute of Technology (Tamil Nadu) and brought to the laboratory. The *A. paniculata* leaves were thoroughly rinsed with deionised water to remove adsorbed dirt and air dried for 2 h and the leaves were further chopped into small pieces and were allowed to shade dried at room temperature (25 °C) for a week. Then the completely dried leaves were powdered and sieved to get fine powder. About 5 g of *A. paniculata* powder was added to 100 mL double distilled water and heated at 90 °C for 10 min. The change in color of the aqueous solution was noticed from watery to thick blackish brown color and the resultant color extract was cooled to room temperature, filtered through Whatmann No. 1 (~11µm) filter paper and was stored at 4 °C until further studies.

Materials Characterization

Crystallographic information of GO, rGO-AP, rGO/ZnO-NC 0.01 M, rGO/ZnO-NC 0.05 M, rGO/ZnO-NC 0.1 M, and ZnO NPs 0.05 M was investigated by X-ray diffraction (XRD) patterns, which were characterized by using a Brucker D8 Advance diffractometer.

The measurements were recorded at room temperature over the range of 2θ from 5° to 80° with a scanning rate of 4°/min using Cu Kα radiation (λ = 1.54 A°) and a step size of 0.02^{o}. Lanthanum hexaboride (LaB_6) was used to calibrate the instrument. ATR-FTIR measurements were carried out to understand the extent of reduction, and the reaction progress of the rGO/ZnO-NC was analysed by using FT-IR spectroscopy in Attenuated Total Reflectance (JASCO ATR-FTIR 4100) mode between a frequency range of 4000-500 cm^{-1}. Scanning electron microscopy (SEM) and energy-dispersive X-ray spectrometry (EDX) analysis were performed using a Carl Zeiss SEM instrument attached to EVO MA 15 camera operating at a voltage of 10.0 keV. The obtained solid dry samples were transferred to a carbon tape held into the SEM sample holder for analysis. Surface morphological images were recorded at diverse magnifications for rGO-AP, rGO/ZnO-NC 0.01 M, rGO/ZnO-NC 0.05 M, rGO/ZnO-NC 0.1 M, and ZnO NPs 0.05 M. Simultaneously, the EDX spectrum measurement was also obtained by zooming the specific surface area on the corresponding solid sample to acquire the elemental composition of the samples. TEM samples were prepared by dispersing the purified dried samples of rGO-AP, rGO/ZnO-NC 0.01 M, rGO/ZnO-NC 0.05 M, rGO/ZnO-NC 0.1 M, and ZnO NPs 0.05 M in double distilled water (0.5 mg mL^{-1}) under ultra-sonication for 1 h. A drop of the aqueous dispersion was placed on a copper grid, which was further left for drying in vacuum.

The selected area electron diffraction (SAED) patterns were examined by using HR-TEM (JEOL JEM 2100) which was driven with an acceleration voltage of 200 kV. X-ray photoelectron spectroscopy (XPS) was applied to analyze the chemical state of C, O, and Zn elements present on the surface of the rGO/ZnO-NC 0.05 M sample. XPS measurements were

carried out using ESCA-3000 VG Scientific UK. The instrument was operated at 150 W using a non-monochromatic Al Kα radiation (1486.6 eV) with a spectral resolution of 0.2 eV at a base pressure of less than 10^{-8} Torr. The C1s and O1s spectra were deconvoluted by using XPS Peak 4.1 software. Raman spectroscopic studies of the prepared GO, rGO-AP, rGO/ZnO-NC 0.05 M and ZnO NPs 0.05 M samples were performed by using a Horiba Jobin Yvon LabRAM ARAMIS under 514.5 nm Ar laser at a power of 0.5 mW and the microscope slide was prepared by dispersing powder sample at room temperature. UV-Vis diffuse reflectance spectra (DRS) were measured in the range of 300-4000 nm using a Jasco V-670 UV-Vis double beam spectrophotometer with an integrating sphere accessory. The powders were pressed into pellets and $BaSO_4$ was used as reference standard for correction of the instrumental background. The reflectance was converted into absorbance according to the Kubelka-Munk function: $F(R)\ \alpha\ K/S = (1-R)^2/2R$, where "K" represents the absorption coefficient, "S" denotes the scattering coefficient and "R" is the diffuse reflectance. Zeta potential and Dynamic light scattering measurements were carried out to study the surface charge as well as the stability and the size distribution of the formed GO, rGO-AP, rGO/ZnO-NC 0.01 M, rGO/ZnO-NC 0.05 M, rGO/ZnO NC- 0.1 M, and ZnO NPs 0.05 M. The measurements were performed using a Horiba Scientific Nanoparticci (SZ-100) instrument and the sample was dispersed (0.5 mg mL^{-1}) in aqueous solution. The capture of the fluorescence microscopic images of all cell lines was performed by using fluorescence microscope (Model No FM-3000, Weswox, Ambala) to confirm the generation of reactive oxygen species after the exposure to the synthesized nanomaterials.

Cell culture:

Two different types of human carcinoma cell lines A549 (adenocarcinomic human alveolar basal epithelial cells) and HCT116 (human colorectal carcinoma cell lines) have

been widely used for cytotoxicity studies. Both cell lines were purchased from NCCS, Pune, (India), and together with one normal cell line Human umbilical cord blood derived mesenchymal stem cells (hMSCs) purchased from PromoCell (Germany) Cells, were cultured in DMEM/(α-MEM) medium supplemented with 10% heat inactivated FBS and 1% penicillin-streptomycin and maintained in 5% CO_2 incubator at 37 °C under 95% humidified atmosphere. For all the cell cultures, the media was changed once every three days. At the confluence of around 80%, cells were trypsinized and used for experiments.

Cytotoxicity Evaluation/MTT assay:

MTT assay was performed using 2-[4, 5-dimethyl-2-thiazolyl]-2 and 5-diphenyl-2-tetrazolium bromide (Sigma, USA) to determine the relative cell viability of A549, HCT116 and hMSCs cell lines. Briefly, 1×10^4 cells/well were seeded in 96-well plates incubated for 24 h and exposed to the synthesized nanocomposites GO, rGO-AP, rGO/ZnO-NC 0.01 M, rGO/ZnO-NC 0.05M, rGO/ZnO-NC 0.1M and ZnO NPs 0.05M at concentrations of 0, 1, 2, 4,6, 8 and 10 ppm to the respective cell line. After 24 h of incubation the culture medium was carefully removed from the wells and wells were washed twice gently with PBS to remove the nanocomposites. The MTT solution (0.5 mg mL^{-1}) was added into the wells along with fresh medium and incubated for 3 h at 37 °C. The formed formazan crystals were solubilized by a stop solution (10% SDS in 0.01 N HCl) according to the manufacturer's protocol (Sigma, USA). The absorbance of coloured solution was measured at 570 nm.

Measurement of Reactive oxygen species (ROS):

The intracellular ROS generation was measured according to the modified method of Wilson *et al*. (2002) by using 2, 7-dichlorofluorescein diacetate (DCFH-DA).[13] DCFH-DA is a non-fluorescence compound that undergoes intracellular deacetylation followed by ROS

mediated oxidation and gets converted to highly fluorescent compound dichlorofluorescein (DCF). Two cancer cell lines (A549, HCT116) and one normal cell line (hMSCs) were treated with GO, rGO-AP, rGO/ZnO-NC 0.01M, rGO/ZnO-NC 0.05M, rGO/ZnO-NC 0.1M and ZnO NPs 0.05M at the concentration of 10 ppm for 24 h. At the end of exposure, the cells were washed twice with Hank's Balanced Salt Solution (HBSS), followed by treating with 10 μM 2′, 7′ CM-H_2DCFDA (Invitrogen, USA) and incubation in the dark at 37°C. Under oxidation this dye emits light at 535 nm, when exposed to an excitation wavelength of 480 nm. The consequent fluorescence images were acquired using a fluorescence microscope (Leica, Germany). The ROS mediated mean fluorescence intensity (MFI) of rGO/ZnO-NCs was quantified by using NIH ImageJ software program. The recorded data (MFI) was expressed as relative fluorescence intensity of dichlorofluorescein (DCF).

Cytotoxicity assay-flow cytometry study:

Apoptosis kit with Annexin V Alexa Fluor® 488 and Propidium Iodide (PI) (Thermofisher, USA) were employed to detect apoptotic and necrotic death cells after exposure to synthesized nanomaterials GO, rGO-AP, rGO/ZnO-NC 0.01M, rGO/ZnO-NC 0.05M, rGO/ZnO-NC 0.1M and ZnO NPs 0.05M. The manual of the kit was strictly followed during all experiments. Cytotoxicity was measured according to the protocol proposed by L.V.K. Reddy *et al.*[14] Briefly, 5×10^4 cells were placed in 6 well plates and incubated for 24 h followed by different NCs treatments to different wells, incubated for 24 h and the apoptosis was measured by using the Dead Cell Apoptosis Kit with Annexin V Alexa Fluor® 488 and Propidium Iodide (PI) (Thermofisher, USA) by following the manufacturers's protocol. Briefly, the cells were trypsinized and centrifuged at 1500 rpm for 5 min. The pellet was washed twice with PBS and re-centrifuged. The pellet was resuspended in 100 μL of 1× Annexin binding buffer and the cells were conjugated with 5 μL of Annexin V Alexa Fluor® 488 and 1 μL of PI. Then cells were mixed gently and incubated for 15 min at room

temperature (RT) in the dark. After the incubation period, 400 µL of 1 × Annexin-binding buffer was added, mixed gently and kept on ice. Data was acquired by flow cytometry (BD FACSCelesta^{TM}, New Jersey, US).

Statistical Analysis

The statistical analysis was performed to check null hypothesis that "there is no significant difference among the data sets" by W.S. Gosset student's t-test considering two tailed paired data sets. As the number of data in each set is 6, the degree of freedom is considered as 10 and the absolute t-value for the two tailed paired data set is considered as 2.23 at the probability (p) of 0.05 levels. According to this hypothesis, if the calculated t-value [absolute (mean1-mean2)/RSQ(variance1/n + variance2/n)] (i.e. p values) is lower than the absolute t-value of 2.23 at p = 0.05, the null hypothesis is accepted, but for higher value it is rejected. Finally, student's t-test was performed to calculate probability value and if the p-value is lower than 0.05, it is considered statistically significant and otherwise data sets are not statistically significant at p = 0.05. Also, we checked skewness of the data sets to check whether normal distribution is followed or not. For that we determined skew value from Excel software and checked whether is higher than 2*(6/n) or not. For higher value we did not perform student's t-test as normal distribution is absent (Table S3A-S3B).

Results

XRD was used to determine the crystalline nature of the as-synthesized GO, rGO-AP, rGO/ZnO-NC (0.01, 0.05, and 0.1M), and ZnO NPs 0.05M. Figure S1A summarizes the XRD patterns of all the six samples. Figure S1A (a) corresponds to the characteristic diffraction peaks of GO and its 2θ values are 10.25°, 42.30° whereas the peak at 10.25° represents to the

d_{002} plane of GO and the other peak located at 2θ = 42.30^o belongs to the hexagonal structure of 100 carbon plane.[1] The d-spacing values of GO are 8.62 A^o and 2.13 A^o respectively (Figure S1A(b)). After the successful reduction of GO to rGO-AP, a remarked decrease in the peak intensity is observed and two new 2θ peaks appear at 25.63^o and 42.96^o corresponding to the d_{002} plane for the rGO-AP with d-spacing values of 3.41 A^o and 2.09 A^o, respectively. The decrease in the d-spacing value was noticed in the case of rGO-AP due to the loss/removal of oxygen containing functional groups from the rGO surface during the reduction process. Patterns c, d, e, f in Figure S1A belong to the as-synthesized rGO/ZnO-NC and pure ZnO NPs with difference in zinc salt precursor concentrations. The relative diffraction peaks can be observed at 2θ values of 31.78°, 34.51°, 36.6°, 47.48°, 56.71°, 62.86°, and 67.87° corresponding to (100), (002), (101), (102), (110), (103), and (112) crystal reflection planes. These patterns obviously show diffraction peaks associated with the standard XRD data file of ZnO (JCPDS standard card no. 79-2205) in wurtzite phase. Increasing Zn^{2+} concentration from rGO/ZnO-NC 0.01M to rGO/ZnO-NC 0.1M results in a gradual increase of the intensity of the ZnO wurtzite crystal plane which implies that the ZnO crystal structure in rGO/ZnO-NC grew larger. The full width half-maximum (FWHM) values of the corresponding diffraction peaks can be calculated by using the Scherrer equation, and their values are 10.47, 13.66, 12.4, and 11.07 nm, respectively.

FT-IR spectroscopy technique was employed successfully to further confirm the removal of oxygen-containing functional groups from the GO surface. Figure S1B denotes the FT-IR spectra of as-synthesized GO, rGO-AP and rGO/ZnO-NCs. The broad absorption peaks recorded at 3377 and 1622 cm^{-1} relate to the O-H group stretching vibration for the absorbed H_2O molecules, and the other peaks located at 1726, 1375 and 1220 cm^{-1} ascribe to the C=O, C–OH and C-O stretching vibrations of epoxy groups respectively. The absorption bands values at 1053, 1093 cm^{-1} correspond to the stretching vibration of C-O alkoxy group.

After the green reduction of GO using aqueous *A. paniculata* leaf extract as suitable reducing and stabilizing agent, the decrease in intensity ratio for oxygen-containing functional groups were almost entirely removed in both pure rGO-AP and rGO/ZnO-NCs. For the pure ZnO NPs one absorption band can be observed at 497 cm^{-1} which is a characteristic stretching vibration for Zn-O. However, red shift in these peak values were noticed at 449, 445 and, 443 cm^{-1} in rGO/ZnO-NCs and it occurs mainly due to interactions existing between ZnO NPs and the residual hydroxyl and epoxy functional groups present on the rGO surface.[2, 3]

Raman spectroscopy is a highly sensitive and non-destructive technique to find out crystallization, electronic structure of the carbon based materials, type of doping and any defects, ordered and disordered crystal structure of graphene.[4] Hence Raman-scattering studies were carried out for the as-prepared GO, rGO-AP, rGO/ZnO-NC 0.05M and ZnO NPs 0.05M and the spectra are shown in Figure S1C. A broad D-band observed at 1328 cm^{-1} is mainly due to the presence of lattice defects in the sp^2-hybridized carbon atoms or in the amorphous carbon. The broad G-band appeared at 1589 cm^{-1} is associated to the highly orientation pattern of sp^2 hexagonal graphitic lattice.[5] These two typical Raman shifts (Figure S1C (a, c)) were observed for GO and rGO/ZnO-NC 0.05M respectively. The increased intensity ratio value of 1.39 was observed for rGO/ZnO-NC 0.05M compared to that of 1.23 for GO, this increase in I_D/I_G value clearly indicates the successful reduction of GO in rGO/ZnO-NC i.e. elimination of oxygen containing moieties from the GO surface and the reestablishment of numerous (sp^2 carbon) conjugated graphene network. In order to understand the green reduction process from GO to rGO-AP, the reaction was performed only in the presence of aqueous *A. paniculata* leaf extract alone by maintaining similar reaction conditions. *A. paniculata* leaf extract is effective to reduce GO to rGO-AP (Figure S1C (b)) and resulting in a I_D/I_G ratio value 1.32. In addition to the presence of G-band and D-bands in the rGO/ZnO-NC and ZnO NPs 0.05M, three more characteristic Raman shifts were

observed at 325, 434 and 570 cm^{-1}, as shown in Figure S1C(c, d). These could be assigned to various vibrational modes of ZnO NPs. The band present at 325 cm^{-1} is attributed to the second order scattering from E_2 high to E_2 low. The peak at 434 cm^{-1} relates to the Raman active ZnO wurtzite hexagonal phase of E_2 (high) optical phonon. Also, the peak positioned at 570 cm^{-1} corresponds to LO phonon of A_1 and E_1 modes of ZnO.[6, 7] The Raman results confirmed that the rGO/ZnO-NC was composed of pure ZnO NPs decorated on graphene nanosheets.

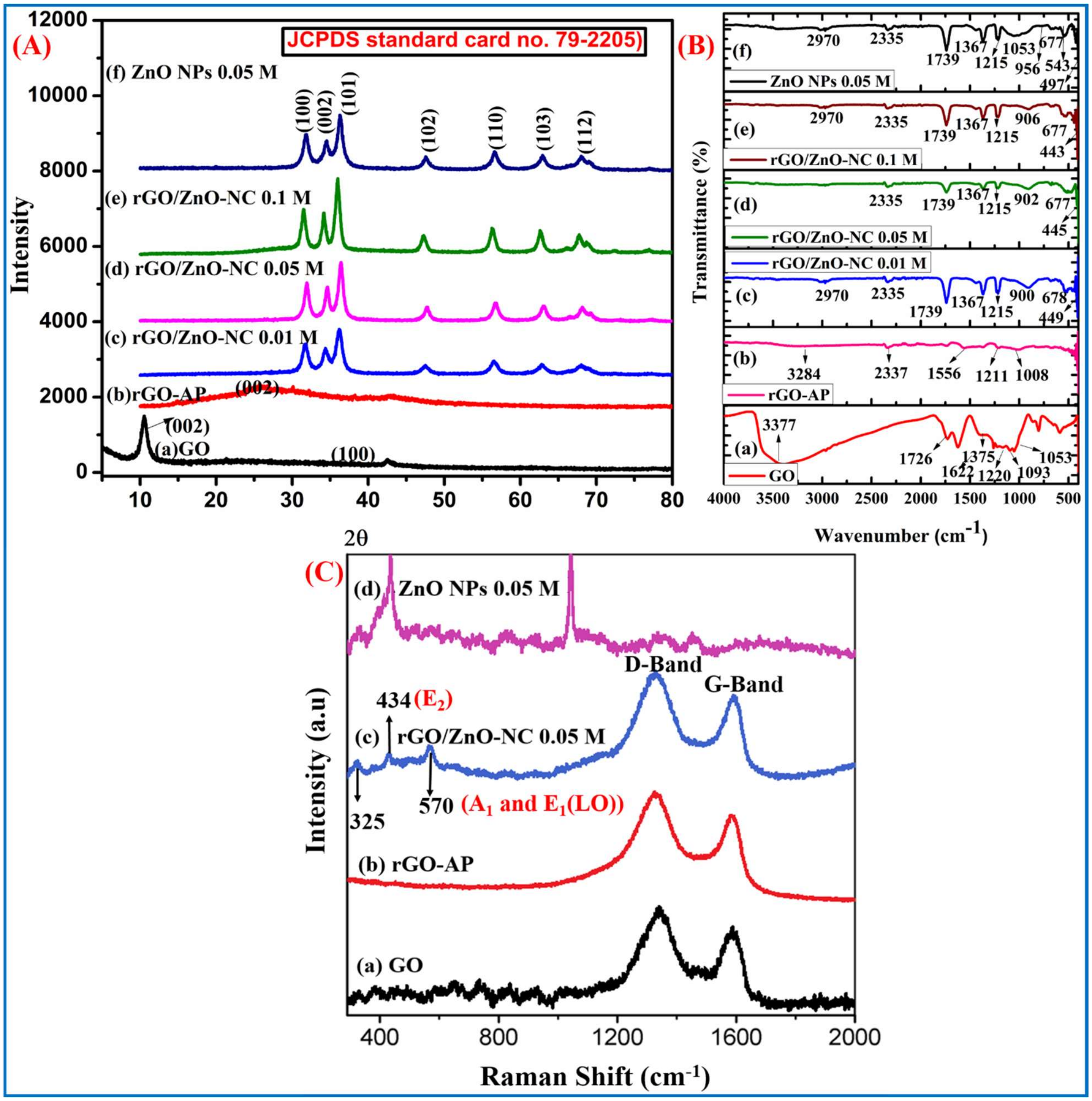

Figure S1: Spectral characterization of different samples. XRD patterns of GO (a), rGO-AP (b), rGO/ZnO-NC 0.01 M (c), rGO/ZnO-NC 0.05 M (d), rGO/ZnO-NC 0.1 M (e), and ZnO NPs 0.05 M (f) (A); FTIR spectra of GO (a), rGO-AP (b), rGO/ZnO-NC 0.01 M (c), rGO/ZnO-NC 0.05 M (d),

rGO/ZnO-NC 0.1 M (e), and ZnO NPs 0.05 M (f) (B); Raman spectra of GO (a), rGO-AP (b), rGO/ZnO-NC 0.05 M (c), and ZnO NPs 0.05 M (d) (C).

The UV-vis absorption spectra of the as-synthesized GO, rGO-AP, rGO/ZnO-NC (0.01M, 0.05M, and 0.1M) and ZnO NPs 0.05M are illustrated in Figure S2. The GO exhibits a characteristic absorption peak at 230 nm, and a small shoulder peak near 300 nm which attributes to C=C aromatic bond ($\pi \rightarrow \pi^*$), and C=O bonds ($n \rightarrow \pi^*$) respectively (Figure S2 A). After the reduction of GO to rGO-AP using aqueous *A. paniculata* leaf extract as reducing stabilizing agent, these peaks red-shift and only one peak appears at 270 nm for rGO-AP corresponding to aromatic C=C bond transition ($\pi \rightarrow \pi^*$) (see Figure S2A (b)). Compared to rGO-AP, rGO/ZnO-NC displays new characteristic absorption peaks at 371 and 378 nm (Figure S2A(c-f)) which resemble the intrinsic absorption of ZnO nanocrystals and these electronic transitions occur between the valence band to the conduction band ($O_{2p} \rightarrow Zn_{3d}$).[8] These results illustrate that the crystalline and impurity suppressed ZnO NPs were successfully dispersed onto the rGO surface for the formation of rGO/ZnO-NC. Figure S2B depicts the Kubelka-Munk remission function plots that represent the relationship of $(\alpha h\upsilon)^2$ vs. photon energy. The band gap energy (Eg) value was calculated based on the equation Eg = he/λ; where 'λ' is the wavelength in nm. The corresponding band gap values obtained from each spectrum are as follows, for pure ZnO NPs 0.05M is 2.9 eV; it decreases to 2.7 and 2.83 eV for the rGO/ZnO-NC 0.01M and 0.05M, respectively. This phenomenon is mainly due to the Zn-O-C chemical bond establishment in the as-synthesized rGO/ZnO-NC.[9]

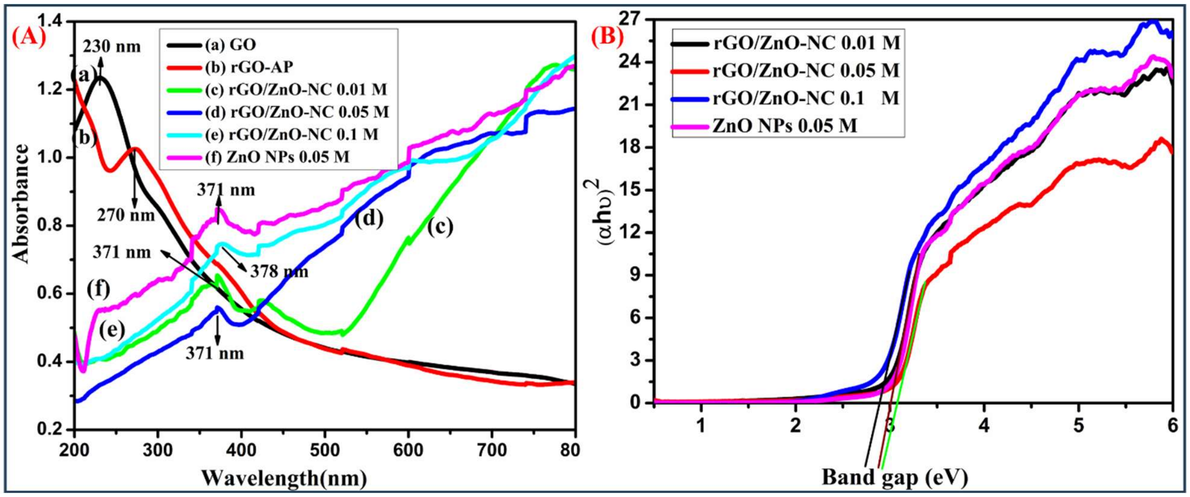

Figure S2. UV-vis spectra of as-prepared nanomaterials GO (a), rGO-AP (b), rGO/ZnO-NC 0.01 M (c), rGO/ZnO-NC 0.05 M (d), rGO/ZnO-NC 0.1 M (e), ZnO NPs 0.05 M (f) (A); the corresponding$(\alpha h\upsilon)^2$ vs. hυ plots of rGO/ZnO-NC (B).

It is well known that nanocomposites tend to agglomerate in solution which shows further effect on their interactions with biological systems. Thus, the characterizations of the as-synthesized samples GO, rGO-AP, rGO/ZnO-NC (0.01 M, 0.05 M, 0.1 M) and ZnO NPs 0.05 M in three different liquid systems such as PBS, culture media and water medium were performed using DLS (dynamic light scattering) analysis. By using this analysis we determined hydrodynamic diameter, zeta potential and polydispersity indices (PI) values of different suspensions as depicted in Table S1 and Figure S3-S8. Despite the excellent colloidal stability exhibited by all the prepared rGO/ZnO-NCs in water system, their stability would vary significantly when transferred into aqueous systems, including biological buffers like Phosphate Buffer Solution (PBS) and DMEM (Cell culture medium). The above results suggest that the exfoliation of all the prepared rGO/ZnO-NCs in water medium was very stable and among all the NCs the highest is recorded for rGO-AP having a zeta potential of -59.5 mV. Minor aggregation is observed for all the functionalized rGO/ZnO-NCs of their dispersion into PBS and cell culture media. This is accompanied by an increase in

hydrodynamic diameters in the cell culture medium when compared to those in PBS and water medium. The hydrodynamic size of GO is maximum (4187.1 nm) followed by rGO/ZnO-NC 0.05M as 4038.3 nm (Table S1). Consistently, the zeta potential is decreased in PBS and cell culture medium (Table S1). As the cell growth media consists of high protein containing biomolecules like serum, albumin, fibrinogen, amino acids, and vitamins, the presence of phosphate impacts on agglomeration and/or precipitation of NPs.[10,11] These findings are useful to identify the reasons for a decrease in colloidal stability upon particle incubations in biological systems such as in PBS and DMEM medium. PI is an autocorrelation function, which corresponds to the ratio of a second moment and square of mean value of logarithm and it represents the extent of size distribution.[12] The rGO/ZnO-NCs suspensions exhibits larger PI values with large ionic strengths i.e., in PBS and DMEM (culture medium) when compared to low ionic strength in water medium (see Table S1).

Table S1: Zeta potential and DLS analysis of GO, rGO-AP, rGO/ZnO-NC 0.01 M, rGO/ZnO-NC 0.05 M, rGO/ZnO-NC 0.1 M and ZnO NPs 0.05 M in different solvent systems such as Phosphate Buffer solution (PBS), Cell culture medium and Water.

Sample	Solvent medium	Zeta (mV)	DLS (nm)	PDI
GO	PBS	-42.3	1601.3	0.301
GO	Cell culture medium	-27.9	4187.1	0.381
GO	Water	-45.8	1323.3	0.264
rGO-AP	PBS	-2.7	4.9	0.833
rGO-AP	Cell culture medium	-14.4	1982.3	0.185
rGO-AP	Water	-59.5	1446.2	0.141
rGO/ZnO-NC 0.01M	PBS	-5.0	0.3	0.305
rGO/ZnO-NC 0.01M	Cell culture medium	-0.6	1056.6	0.107
rGO/ZnO-NC 0.01M	Water	-37.5	54.1	2.127
rGO/ZnO-NC 0.05M	PBS	-12.5	2409.6	0.447
rGO/ZnO-NC 0.05M	Cell culture medium	5.0	4038.3	0.381
rGO/ZnO-NC 0.05M	Water	-41.2	3.2	0.316
rGO/ZnO-NC 0.1M	PBS	2.1	2280.8	0.213
rGO/ZnO-NC 0.1M	Cell culture medium	-4.1	2547.3	0.246
rGO/ZnO-NC 0.1M	Water	-36.3	916.8	0.183
ZnO NPs 0.05M	PBS	-12.0	1345.1	0.260
ZnO NPs 0.05M	Cell culture medium	-5.8	2708.5	0.249
ZnO NPs 0.05M	Water	-24.6	4232.5	0.873

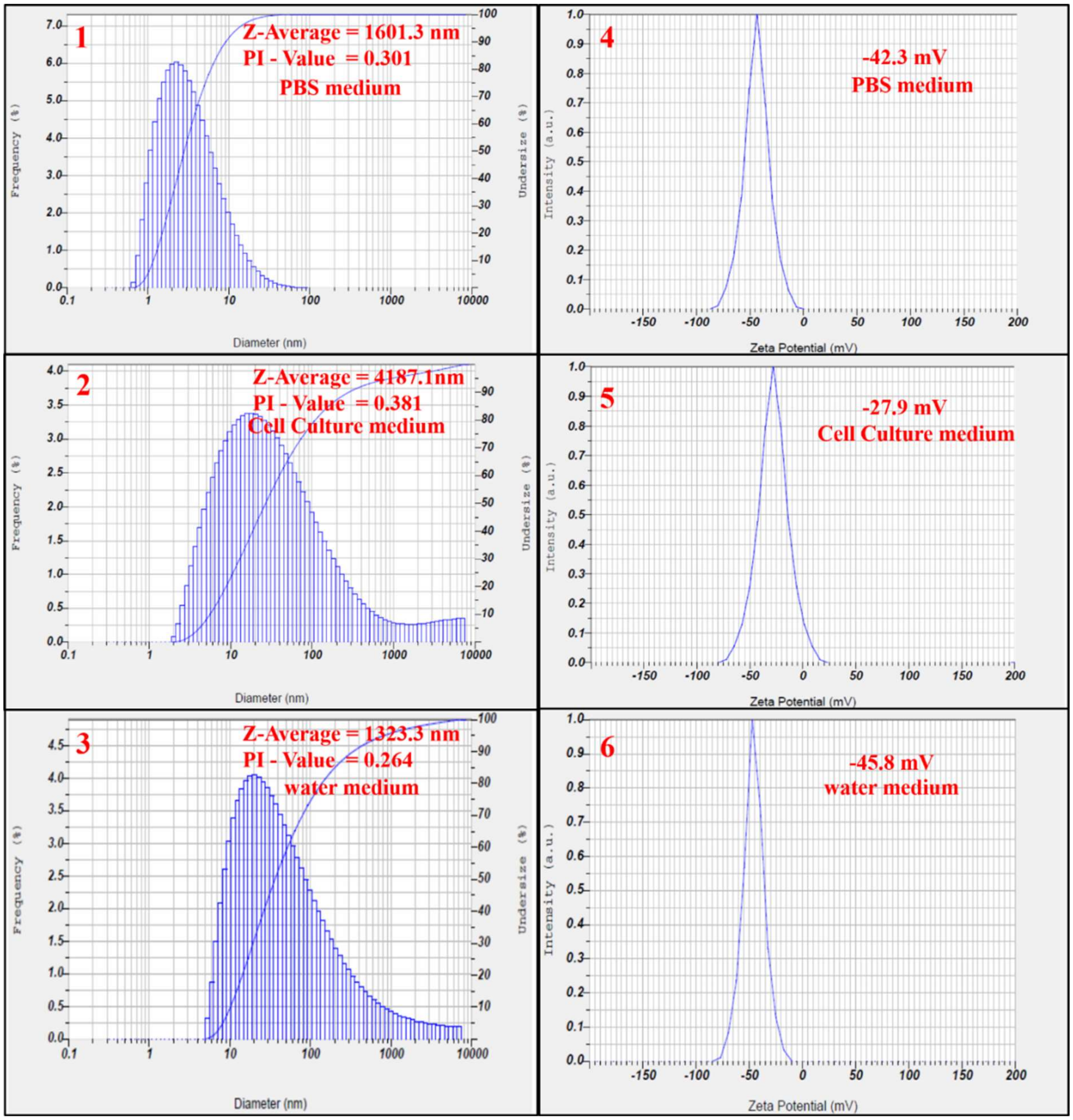

Figure S3. Hydrodynamic size distribution and Zeta potential of GO (0.5 mg mL^{-1}) in PBS medium (1, 4), Cell culture medium (2, 5) and Water medium (3, 6) were measured by DLS at room temperature.

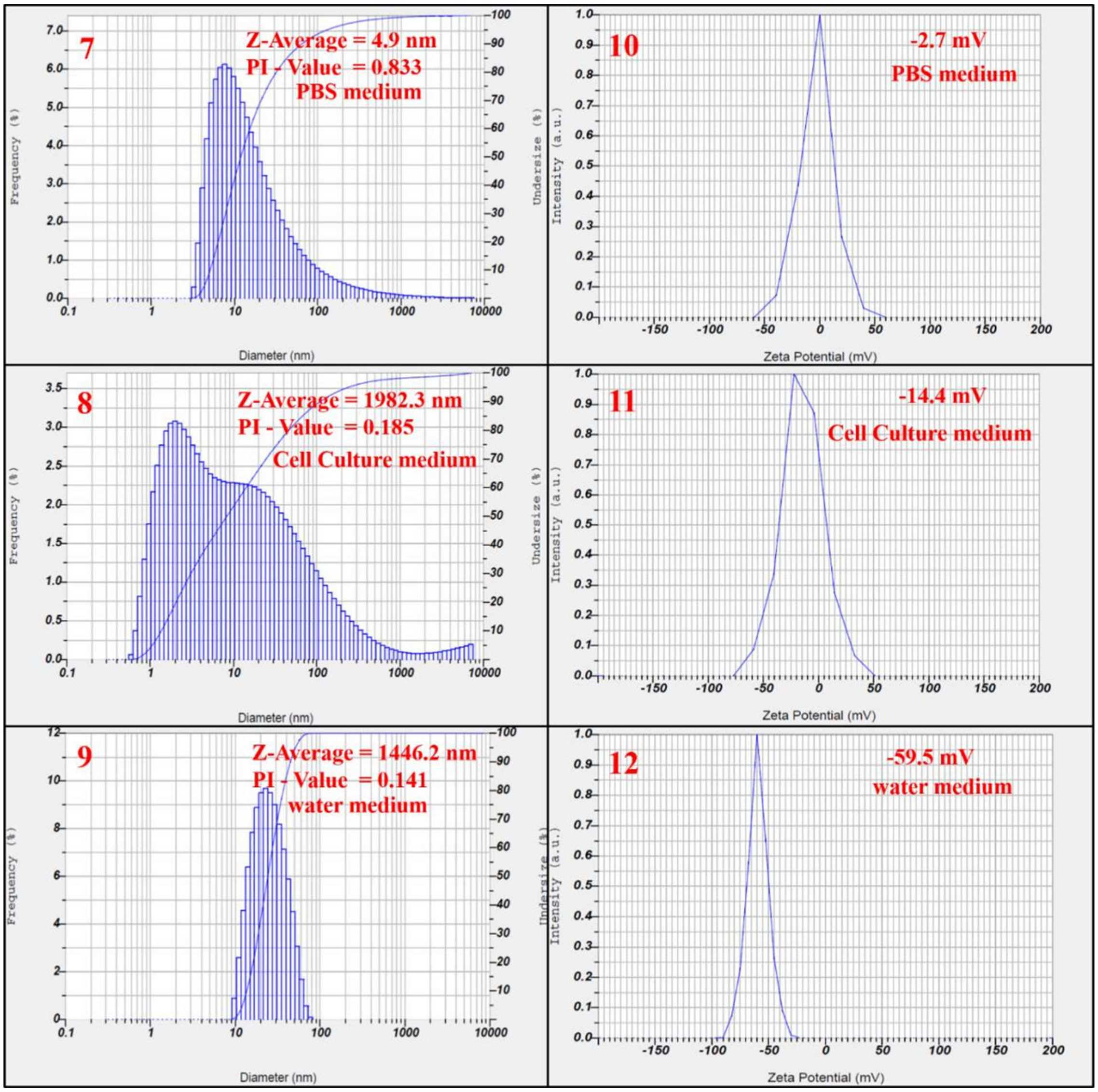

Figure S4. Hydrodynamic size distribution and Zeta potential of rGO-AP (0.5 mg mL^{-1}) in PBS medium (7, 10), Cell culture medium (8, 11) and Water medium (9, 12) were measured by DLS at room temperature.

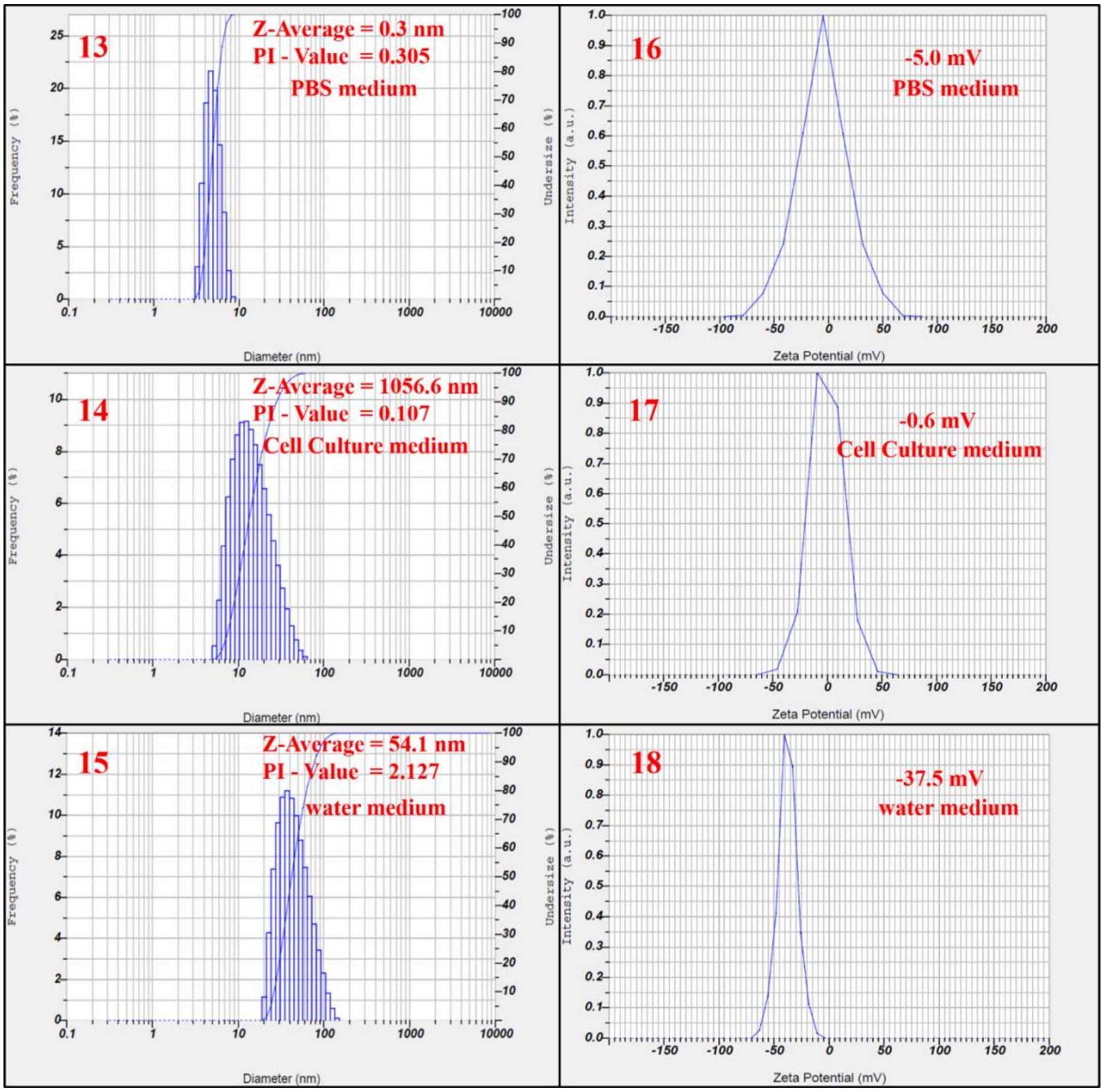

Figure S5. Hydrodynamic size distribution and Zeta potential of rGO/ZnO-NC 0.01M (0.5 mg mL^{-1}) in PBS medium (13, 16), Cell culture medium (14, 17) and Water medium (15, 18) were measured by DLS at room temperature.

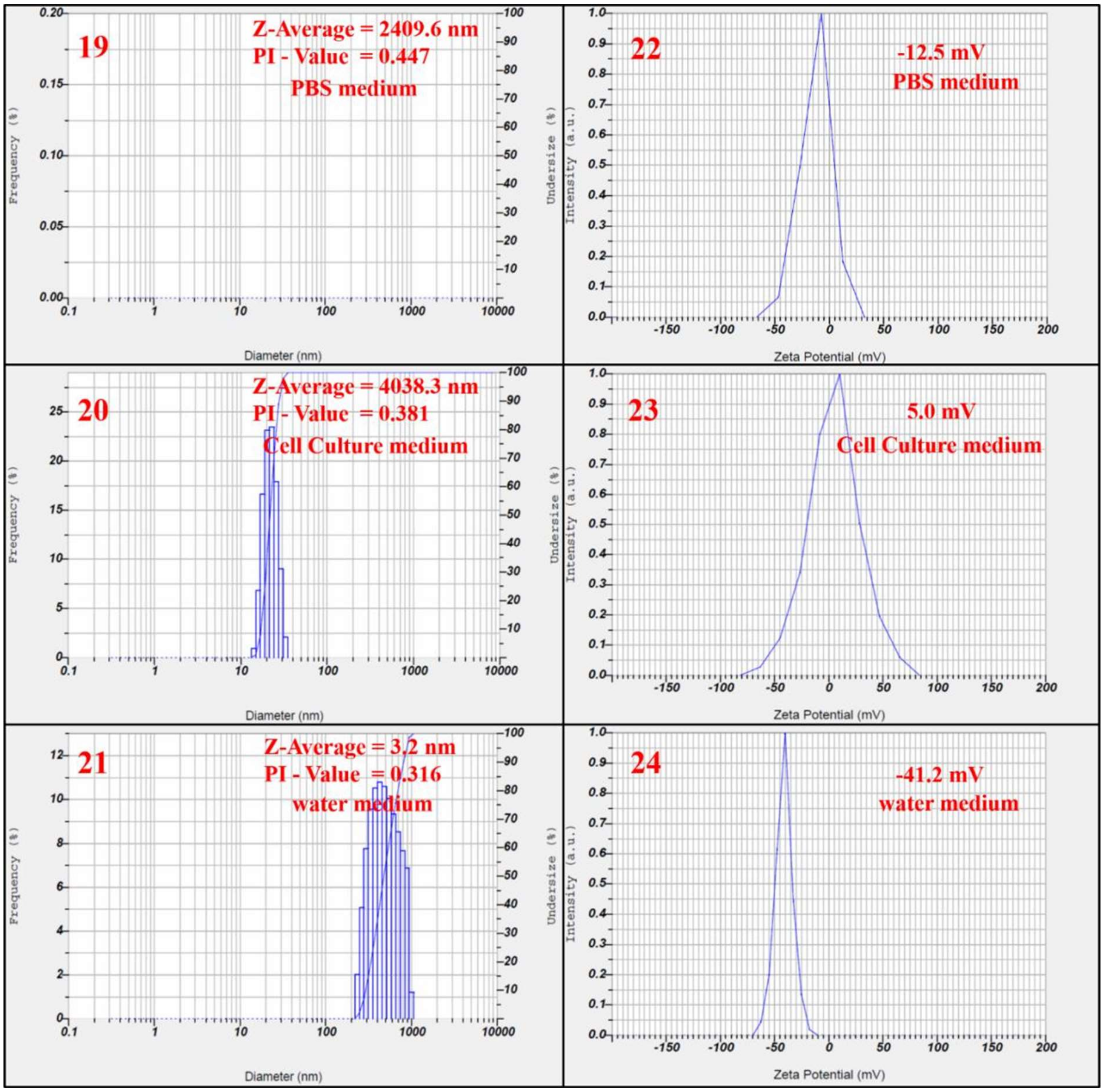

Figure S6. Hydrodynamic size distribution and Zeta potential of rGO/ZnO-NC 0.05M (0.5 mg mL^{-1}) in PBS medium (19, 22), Cell culture medium (20, 23) and Water medium (21, 24) were measured by DLS at room temperature.

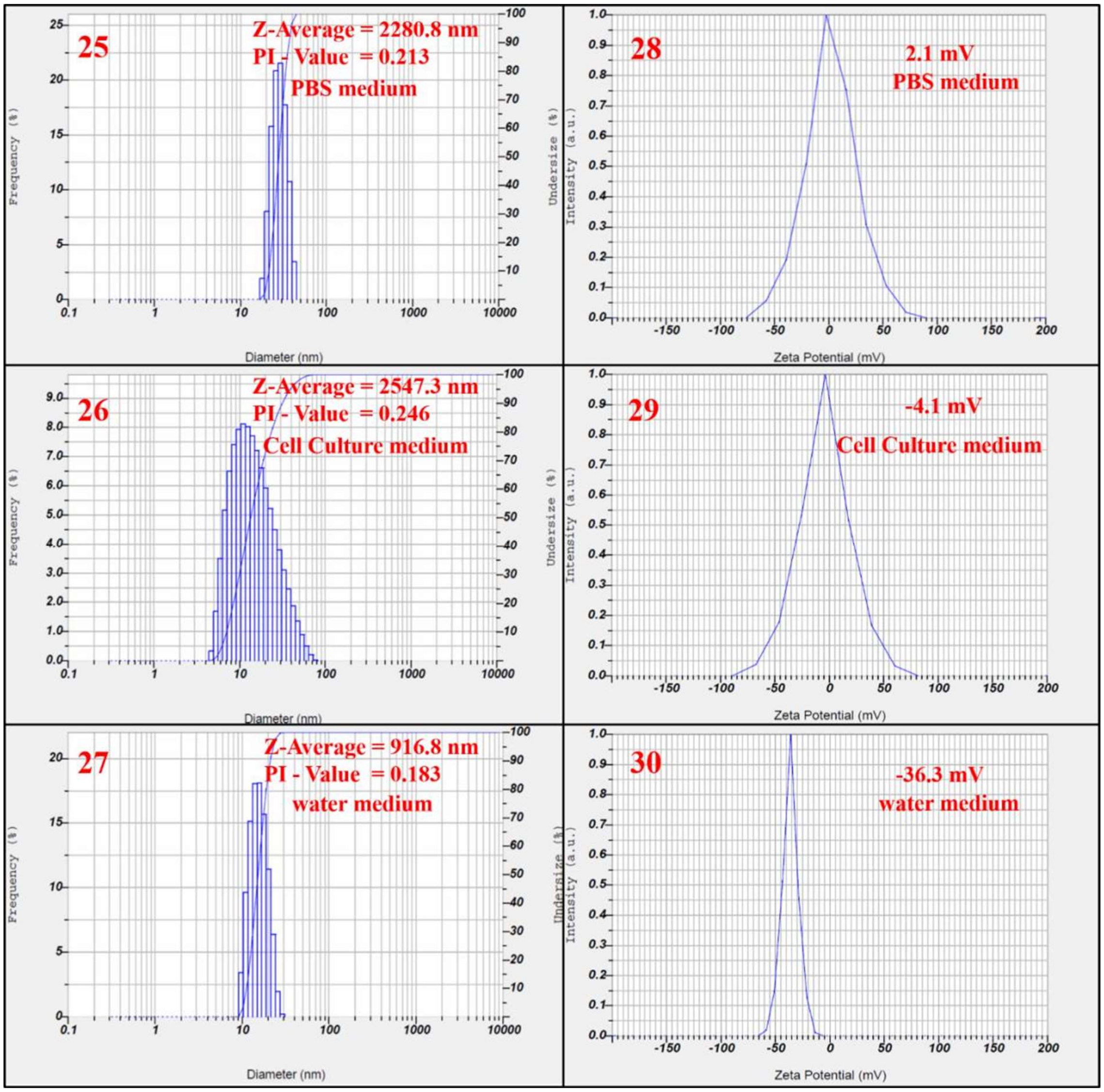

Figure S7. Hydrodynamic size distribution and Zeta potential of rGO/ZnO-NC 0.1M (0.5 mg mL^{-1}) in PBS medium (25, 28), Cell culture medium (26, 29) and Water medium (27, 30) were measured by DLS at room temperature.

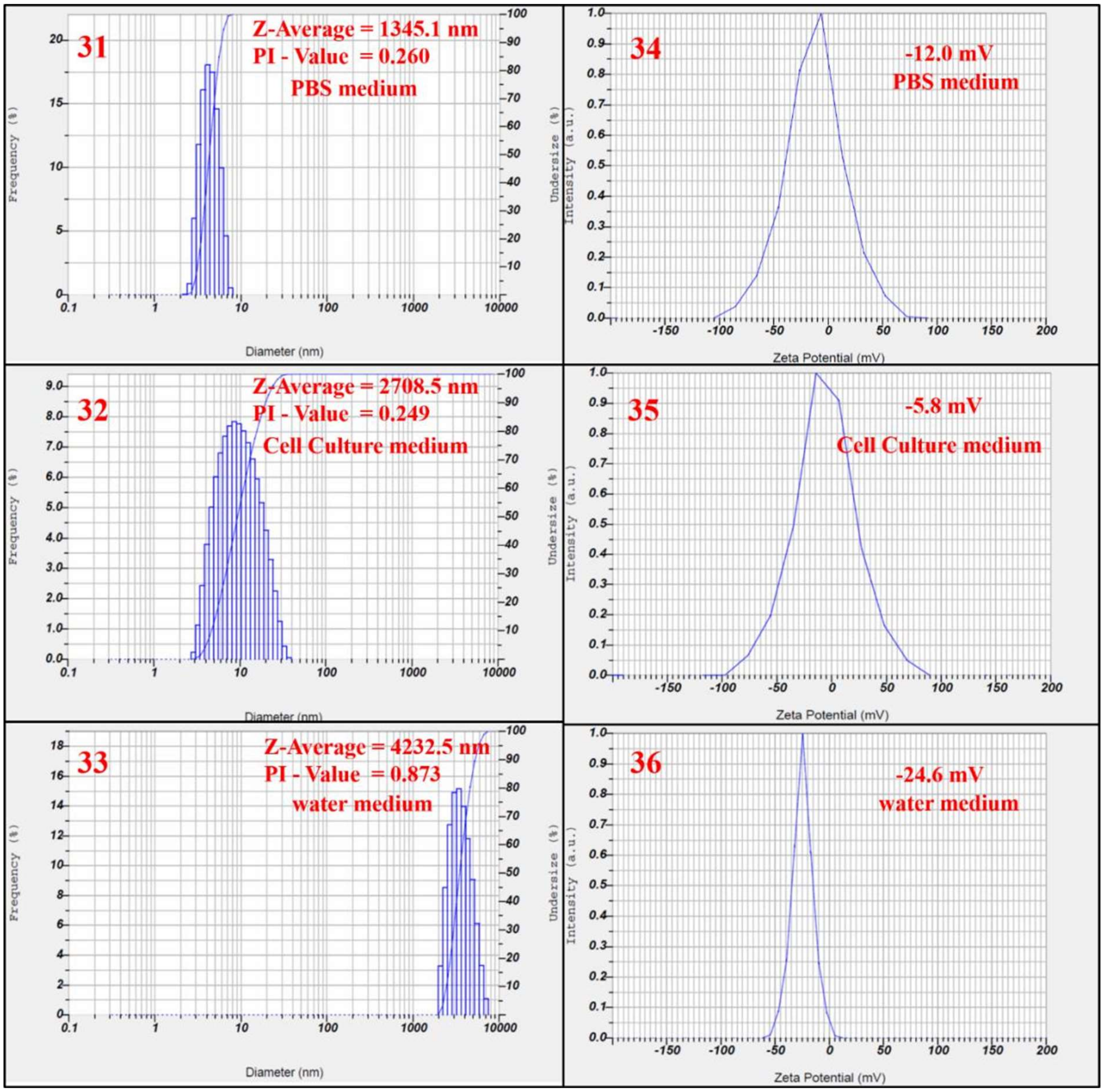

Figure S8. Hydrodynamic size distribution and Zeta potential of ZnO NPs 0.05M (0.5 mg mL^{-1}) in PBS medium (31, 34), Cell culture medium (32, 35) and Water medium (33, 36) were measured by DLS at room temperature.

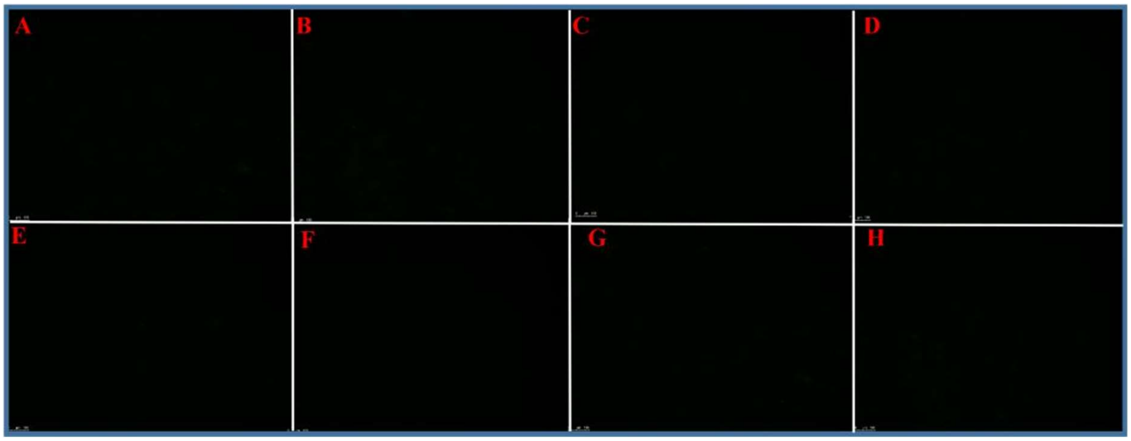

Figure S9. Representative fluorescence microscopic images of ROS generation for HCT116 cancerous cell line; Negative control (A), GO (B), rGO-AP (C), rGO/ZnO-NC 0.01M (D), rGO/ZnO-NC 0.05M (E), rGO/ZnO-NC 0.1M (F), ZnO NPs 0.05M (G), Positive control drug (Cisplatin) (H).

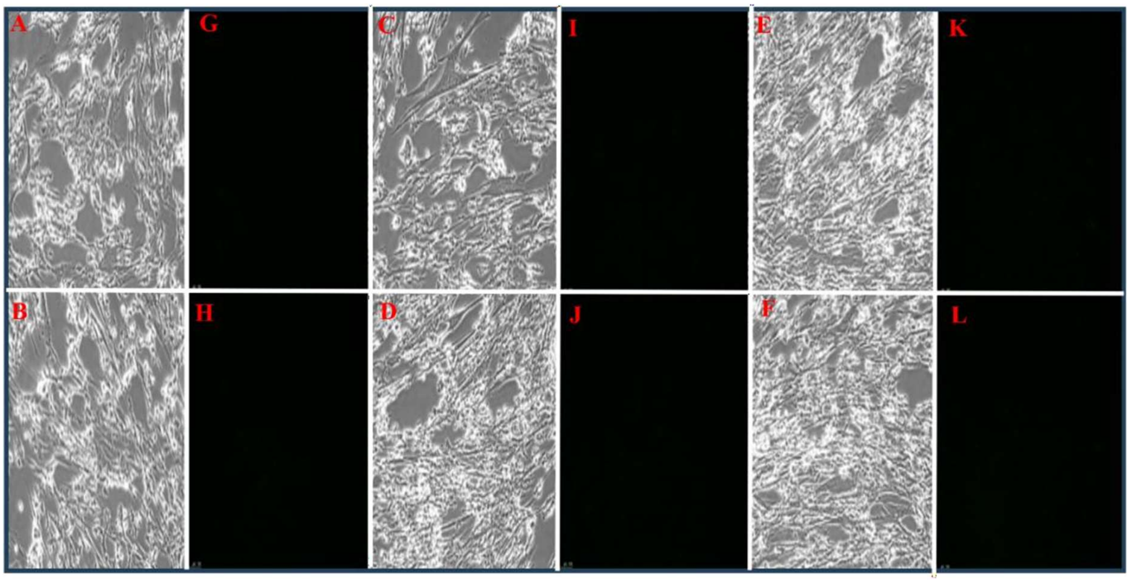

Figure S10: Representative fluorescence microscopic images of ROS generation for hMSCs normal cell line; GO (G), rGO-AP (H), rGO/ZnO-NC 0.01M (I), rGO/ZnO-NC 0.05M (J), rGO/ZnO-NC 0.1M (K), ZnO NPs 0.05M (L). Images are representative of 3 independent experiments. Quantification of the mean fluorescence intensity is done using Image J from 3 images from each run from different groups. Data is average ± SE of 3 independent runs done in triplicate wells in each run (I). $^*p < 0.05$.

Table S2: IC_{50} Values (µg L^{-1}) of nanomaterials GO, rGO-AP, rGO/ZnO-NC 0.01M, rGO/ZnO-NC 0.05M, rGO/ZnO-NC 0.1M, and ZnO NPs 0.05M for human cancerous cell lines.

Sample	Incubation time	A549 Cell line	HCT116 Cell line
GO	24 h	3.6670	3.1064
rGO-AP	24 h	3.2987	1.4454
rGO/ZnO-NC 0.01M	24 h	0.3461	0.0515
rGO/ZnO-NC 0.05M	24 h	0.5634	0.2831
rGO/ZnO-NC 0.1M	24 h	0.8274	0.5843
ZnO NPs 0.05M	24 h	2.5460	1.5526

Table S3: The ROS quantified mean fluorescence intensity (MFI) data values for Negative control, GO, rGO-AP, rGO/ZnO-NC 0.01M, rGO/ZnO-NC 0.05M, rGO/ZnO-NC 0.1M, ZnO NPs 0.05M, and Positive control drug (Cisplatin) for human cancerous cell lines.

Sample	A549 Cell line (MFI) Values	HCT116 Cell line (MFI) Values
Negative control	0.39	1.82
GO	2.82	14.5
rGO-AP	3.36	14.59
rGO/ZnO-NC 0.01M	5.29	22.61
rGO/ZnO-NC 0.05M	3.94	16.37
rGO/ZnO-NC 0.1M	3.67	10.62
ZnO NPs 0.05M	2.38	7.95
Positive control drug (Cisplatin)	3.15	9.29

Table S4: Data obtained from FITC-conjugated annexin-V and PI staining assay for A549 cells treated with 10 μg L^{-1} each of the respective samples i.e. Negative control, Positive control drug (Cisplatin), GO, rGO-AP, rGO/ZnO-NC 0.01M, rGO/ZnO-NC 0.05M, rGO/ZnO-NC 0.1M, and ZnO NPs 0.05M

Sample	Q1 (%)	Q2 (%)	Q3 (%)	Q4 (%)
Negative control	0	0	99.3	0.7
Positive control drug (Cisplatin)	0	22.7	1.3	76.0
GO	1.6	42.4	16.1	40
rGO-AP	0.4	50.1	13.6	35.9
rGO/ZnO-NC 0.01M	0	41.5	0.9	57.7
rGO/ZnO-NC 0.05M	0	45.1	2.4	52.5
rGO/ZnO-NC 0.1M	0	48.1	4.6	47.2
ZnO NPs 0.0 M	0.6	58.0	9.2	32.2

Abbreviations: Q1: necrotic cells, Q2: late apoptotic, Q3: region denotes live cells, and Q4: apoptotic.

Table S5: Data obtained from FITC-conjugated annexin-V and PI staining assay for HCT116 cells treated with 10 µg L^{-1} each of the respective samples i.e. Negative control, Positive control drug (Cisplatin), GO, rGO-AP, rGO/ZnO-NC 0.01M, rGO/ZnO-NC 0.05M, rGO/ZnO-NC 0.1M, and ZnO NPs 0.05 M

Sample	Q1 (%)	Q2 (%)	Q3 (%)	Q4 (%)
Negative control	0.4	0.1	99.2	0.3
Positive control drug (Cisplatin)	0	56.0	0.4	43.6
GO	0.2	26.0	6.4	67.4
rGO-AP	0	8.2	2.1	89.7
rGO/ZnO-NC 0.01 M	0.1	96.4	0.1	3.4
rGO/ZnO-NC 0.05 M	0	94.5	0.1	5.4
rGO/ZnO-NC 0.1 M	0	91.1	0.6	8.3
ZnO NPs 0.05 M	0.1	54.2	2.5	43.2

Abbreviations: Q1: necrotic cells, Q2: late apoptotic, Q3: region denotes live cells, and Q4: apoptotic.

Table S6. Statistical analysis of data sets for significant evaluation (A) A549 cell lines (B) HCT116 cell lines (C) hMSCs cells.

**Table 6A**	**1**	**2**	**3**	**4**	**5**	**6**
	GO	rGO-AP	rGO/ZnO-NC 0.01 M	rGO/ZnO-NC 0.05 M	rGO/ZnO-NC 0.1 M	ZnO NPs 0.05 M
Concentration (ppm)	%Viability	%Viability	%Viability	%Viability	%Viability	%Viability
1	41.7	39.9	21.8	23.1	25.2	35.5
2	40.2	38.7	20.5	22.5	24.1	34.8
4	38.6	36.7	19.7	21.6	23.7	33.3
6	37.4	35.9	18.2	20.7	22.1	32.4
8	36.8	34.7	16.7	18.9	20.5	31.7
10	35.4	33.8	14.8	17.3	19.4	30.2
Mean	38.35	36.62	18.62	20.68	22.5	32.98
SD	2.31	2.33	2.58	2.22	2.24	1.97
Variance	5.34	5.45	6.65	4.92	5.01	3.89
Variance/n	0.89	0.91	1.11	0.82	0.84	0.65
Sum of variance/n		1.80	2.00	1.71	1.73	1.54
TSQ (1 vs. others)		1.341640787	1.414213562	1.307669683	1.315294644	1.240967365
t-value		1.29	13.95	13.51	12.05	4.32
df	10	10	10	10	10	
critical t-value (@p=0.05)		2.23	2.23	2.23	2.23	2.23
SE/RSD	0.94	0.95	1.05	0.91	0.91	0.81
p value (t-test)		1.1E-05	6.3E-09	2.2E-08	1.9E-08	0.000000720
			2vs.3	2 vs. 4	2 vs. 5	2 vs. 6
			1.4E-08	3.3E-08	2.8E-06	8.06574E-06
				3 vs. 4	3 vs. 5	3 vs. 6
				9.7E-05	2.8E-06	6.39808E-08
					4 vs.5	4 vs. 6
					3.4E-05	2.83377E-08
						5 vs. 6
						8.45514E-08
skew	0.33	0.35	-0.39	-0.65	-0.33	-2.48

**Table 6B**	1	2	3	4	5	6
	GO	rGO-AP	rGO/ZnO -NC 0.01M	rGO/ZnO -NC 0.05M	rGO/ZnO -NC 0.1M	ZnO NPs 0.05M
Concentration (ppm)	%Viability	%Viability	%Viability	%Viability	%Viability	%Viability
1	38.4	29.4	19.3	21.1	23.7	30.7
2	37.3	28.2	18.6	20.2	22.5	28.7
4	36.6	27.6	17.4	19.5	21.6	27.4
6	35.1	25.4	16.5	18.3	20.3	26.6
8	34.5	24.9	14.9	16.9	18.8	25.4
10	32.7	23.1	12.8	14.6	17.8	24.5
Mean	35.77	26.43	16.58	18.43	20.78	27.22
SD	11.41	8.02	4.57	5.18	5.95	8.28
Variance	130.15	64.28	20.88	26.82	35.42	68.54
Variance/n	21.69	10.71	3.48	4.47	5.90	11.42
Sum of variance/n		32.41	25.17	26.16	27.60	33.12
TSQ (1 vs. others)		5.692978131	5.0169712	5.114684741	5.253570215	5.754997828
t-value		1.64	3.82	3.39	2.85	1.49
df	10	10	10	10	10	10
critical t-value (@p=0.05)		2.23	2.23	2.23	2.23	2.23
SE/RSD		3.27	1.87	2.11	2.43	3.38
		1 vs. 2	1 vs. 3	1 vs. 4	1 vs. 5	1 vs. 6
p value (t-test)		1.23538E-08	2.6818E-09	2.79873E-09	1.85986E-09	2.60337E-07
			2 vs. 3	2 vs. 4	2 vs. 5	2 vs. 6
			9.1597E-08	1.69195E-07	5.14789E-06	0.027663948
				3 vs. 4	3 vs. 5	3 vs. 6
				1.65376E-06	3.03269E-06	3.40562E-07
					4 vs. 5	4 vs. 6
					7.4612E-05	1.2171E-06
						5 vs. 6
						2.61524E-07
skew	-2.49	-2.26	-1.52	-1.78	-2.02	-2.30

**Table 6C**	1	2	3	4	5	6	7	8
	Plant Extract	GO	rGO-AP	rGO/ZnO -NC 0.01M	rGO/ZnO-NC 0.05M	rGO/ZnO-NC 0.1M	ZnO NPs 0.05M	cisplatin
Concentration (ppm)	%Viability	%Viability	%Viability	%Viability	%Viability	%Viability	%Viability	%Viability
	0.228	0.206	0.206	0.202	0.179	0.234	0.22	0.127
	0.206	0.224	0.217	0.126	0.199	0.161	0.173	0.094
	0.202	0.212	0.217	0.211	0.187	0.203	0.233	0.062
Mean	0.212	0.214	0.213	0.180	0.188	0.199	0.209	0.094
StDev	1.39	1.39	1.39	1.41	1.41	1.40	1.40	1.45
Variance	1.94	1.94	1.94	1.99	1.98	1.96	1.95	2.11
Variance /n	0.65	0.65	0.65	0.66	0.66	0.65	0.65	0.70
Sum of Variance /n	1.29		1.29	1.31	1.31	1.30	1.30	1.35
TSQ (2 vs. others)	1.13578167		1.1357 82	1.1445 52	1.144552314	1.140175425	1.14017 54	1.161895004
t-value	0.38		0.38	0.34	0.35	0.36	0.37	0.27
df	4.00	4.00	4.00	4.00	4.00	4.00	4.00	4.00
Critical t-value (@p=0.05)	2.78	2.78	2.78	2.78	2.78	2.78	2.78	2.78
P value (t-test)			8.7E-01	3.9E-01	6.7E-04	6.3E-01	0.837661723	0.029811225
P value (t-test)	96.80	97.72	97.41	82.04	86.00	91.02	95.28	43.07

References

1. Zhang, Y.; Tang, Z.R.; Fu, X.; Xu, Y.J. TiO_2− graphene nanocomposites for gas-phase photocatalytic degradation of volatile aromatic pollutant: is TiO2− graphene truly different from other TiO_2− carbon composite materials?. *ACS Nano*. **2010**, *4*: 7303-7314.

2. Luo, Q.P.; Yu, X.Y.; Lei, B.X.; Chen, H.Y.; Kuang, D.B.; Su, C.Y. Reduced graphene oxide- hierarchical ZnO hollow sphere composites with enhanced photocurrent and photocatalytic activity. *J. Phys. Chem. C*. **2012**, *116*, 8111-8117.

3. Zhang, Q.; Tian, C.; Wu, A.; Tan, T.; Sun, L.; Wang, L.; Fu, H. A facile one-pot route for the controllable growth of small sized and well-dispersed ZnO particles on GO-derived graphene. *J. Mater. Chem*. **2012**, *22*: 11778-11784.

4. Wang, G.; Shen, X.; Yao, J.; Park, J. Graphene nanosheets for enhanced lithium storage in lithium ion batteries. *Carbon*. **2009**, *47*, 2049-2053.

5. Fan, H.; Zhao, X.; Yang, J.; Shan, X.; Yang, L.; Zhang, Y.; Li, X.; Gao, M. ZnO–graphene composite for photocatalytic degradation of methylene blue dye. *Catal. Commun*. **2012**, *29*, 29-34.

6. Youn, C.J.; Jeong, T.S.; Han, M.S.; Kim, J.H. Optical properties of Zn-terminated ZnO bulk. *J. Cryst. Growth*. **2004**, *261*, 526-532.

7. Scott, J.F. UV resonant Raman scattering in ZnO. *Phys. Rev. B*. 1970, *2*,1209.

8. Yu, H.; Yu, J.; Cheng, B.; Zhou, M. Effects of hydrothermal post-treatment on microstructures and morphology of titanate nanoribbons. *J. Solid State Chem*. **2006**, *179*,349-354.

9. Zhang, H.; Lv, X.; Li, Y.; Wang, Y.; Li, J. P25-graphene composite as a high performancephotocatalyst. *ACS nano*. **2009**, *4*, 380-386.

10. Deng, X.; Luan, Q.; Chen, W.; Wang, Y.; Wu, M.; Zhang, H.; Jiao, Z. Nanosized zinc oxide particles induce neural stem cell apoptosis. *Nanotech*. **2009**, *20*, 115101.

11. Reed, R.B.; Ladner, D.A.; Higgins, C.P.; Westerhoff, P.; Ranville, J.F. Solubility of nano-zinc oxide in environmentally and biologically important matrices. *Environ Toxicl Chem*. **2012**, *31*, 93-99.

12. Xu, R. Particle Characterization: Light Scattering Methods, Kluwer Academic Publishers, Norwell (MA), **2001**, Chapter 5.

13. Wilson, M.R.; Lightbody, J.H.; Donaldson, K.; Sales, J.; Stone, V. Interactions between ultrafine particles and transition metals in vivo and in vitro. *Toxicol Appl Pharm*. **2002**, *184*, 172-179.

14. Reddy, L.V.K.; Sen, D. dadle enhances viability and anti-inflammatory effect of human Mscs subjected to ‘serum free’apoptotic condition in part via the Dor/pi3k/akt pathway. *Life Sci*. **2017**, 191, 195-204.
